# Genome-Wide Analysis of the Phosphoinositide Kinome from Two Ciliates Reveals Novel Evolutionary Links for Phosphoinositide Kinases in Eukaryotic Cells

**DOI:** 10.1371/journal.pone.0078848

**Published:** 2013-11-11

**Authors:** George Leondaritis, John Siokos, Irini Skaripa, Dia Galanopoulou

**Affiliations:** 1 Department of Pharmacology, Medical School, University of Thessaly, Larissa, Greece; 2 Laboratory of Biochemistry, Department of Chemistry, University of Athens, Athens, Greece; Laboratoire de Biologie du Développement de Villefranche-sur-Mer, France

## Abstract

**Background:**

The complexity of phosphoinositide signaling in higher eukaryotes is partly due to expansion of specific families and types of phosphoinositide kinases (PIKs) that can generate all phosphoinositides via multiple routes. This is particularly evident in the PI3Ks and PIPKs, and it is considered an evolutionary trait associated with metazoan diversification. Yet, there are limited comprehensive studies on the PIK repertoire of free living unicellular organisms.

**Methodology/Principal Findings:**

We undertook a genome-wide analysis of putative PIK genes in two free living ciliated cells, *Tetrahymena* and *Paramecium*. The *Tetrahymena thermophila* and *Paramecium tetraurelia* genomes were probed with representative kinases from all families and types. Putative homologs were verified by EST, microarray and deep RNA sequencing database searches and further characterized for domain structure, catalytic efficiency, expression patterns and phylogenetic relationships. In total, we identified and characterized 22 genes in the *Tetrahymena thermophila* genome and 62 highly homologues genes in *Paramecium tetraurelia* suggesting a tight evolutionary conservation in the ciliate lineage. Comparison to the kinome of fungi reveals a significant expansion of PIK genes in ciliates.

**Conclusions/Significance:**

Our study highlights four important aspects concerning ciliate and other unicellular PIKs. First, ciliate-specific expansion of PI4KIII-like genes. Second, presence of class I PI3Ks which, at least in *Tetrahymena*, are associated with a metazoan-type machinery for PIP_3_ signaling. Third, expansion of divergent PIPK enzymes such as the recently described type IV transmembrane PIPKs. Fourth, presence of possible type II PIPKs and presumably inactive PIKs (hence, pseudo-PIKs) not previously described. Taken together, our results provide a solid framework for future investigation of the roles of PIKs in ciliates and indicate that novel functions and novel regulatory pathways of phosphoinositides may be more widespread than previously thought in unicellular organisms.

## Introduction

Phosphoinositides (PIs) represent a group of membrane phospholipids that play critical roles in regulating most aspects of eukaryotic cell physiology. Their production from phosphatidylinositol (PtdIns) proceeds through a set of specific PI kinases (PIKs) [1]–[4]. A fundamental aspect of PI function in eukaryotic cells is that different PIs can be generated in a time- and context-dependent manner on distinct membrane subcompartments. Pivotal to this aspect is the presence of multiple PIKs that can generate the same PI and are often co-expressed. A consequence is that membrane subcompartments can be tagged with specific PIs. This, in turn, recruits cytosolic protein effectors to the membrane in order for a specific trafficking or signaling step to take place. Accordingly, PIs can regulate membrane trafficking pathways as is the case of PtdIns3P in endosomal/phagosomal trafficking and PtdIns4P in Golgi secretory function [5]. Furthermore, PIs can serve directly as precursors of water-soluble or lipid second messengers. Two well-established examples are the receptor-activated PI-specific phospholipase C (PI-PLC) and PI 3-kinases class I that utilize PtdIns(4,5)P_2_ to produce Ins(1,4,5)P_3_ and PtdIns(3,4,5)P_3_, respectively, in mammalian cells [6].

Each PIK can be classified into one of three separate families, the PI3K/PI4K family (which includes PI3Ks, type III PI4Ks and some closely related protein kinases), the type II PI4K family and the PIPK family [7]. Members of the PI3K/PI4K III family have retained a distant but recognizable homology to protein kinases. On the contrary, PIPKs occupy a distinct branch of the atypical kinase group [8] and may constitute one of the most divergent families when compared to the rest of eukaryotic protein kinases [9]. Nevertheless, all PIKs bear the three catalytic residues, namely one Lys and two Asp residues in motifs structurally analogous to the VAIK, HRD and DFG motifs in the catalytic domain of protein kinases [9]. Interestingly, protein pseudo-kinases are frequent in the human genome and are characterized by the lack of at least one of these conserved catalytic residues [10], [11]. So far, however, no such pseudo-PIK has been described.

PIKs are ubiquitous eukaryotic enzymes that have been extensively studied in metazoa and the yeast *S. cerevisiae* [6]. Yet, there is limited information on the PIK repertoire of the vast majority of unicellular eukaryotes. A recent analysis established the presence of four or five core eukaryotic PIKs [7]. Unfortunately, few protists were included in this analysis and those were primarily parasitic organisms. Taking into account that the vast knowledge of PI metabolism and functions comes from studies in the crown group of eukaryotes, the analysis of free-living protists may reveal novel evolutionary paths that have been undertaken during evolution. Amongst free-living protists, ciliates represent an exceptional paradigm of cellular elaboration and complex architecture in a single cell. Ciliates are part of the alveolates, one of the most firmly established protist assemblages [12]. *Tetrahymena thermophila* and *Paramecium tetraurelia* are two well-studied ciliates with completed sequenced genomes [13], [14] that have contributed significantly to diverse fields of molecular and cell biology, including membrane trafficking [15]–[20]. PIs have been studied in *Tetrahymena* and *Paramecium* [21]–[23] and there are reports on their involvement in cellular differentiation, enzyme secretion and osmotic stress [24]–[26], [23]. Recent studies have also shown an expansion of PI-PLC genes and Ins(1,4,5)P_3_-receptor channels (IP3R) suggesting the functional coupling of PI-PLC activity and Ca^2+^ regulation in ciliates [27]–[31]. This is a prominent aspect of PI signaling in metazoa that has been elusive in fungi and plants [6].

In this report we have undertaken a genome-wide survey of putative PIK genes in *Tetrahymena* and *Paramecium*. We have identified and characterized a total of 22 putative PIKs in the *Tetrahymena thermophila* genome and an expanded set of 62 PIKs in *Paramecium tetraurelia.* The latter reflects the fact that *P. tetraurelia* has undergone at least two rounds of whole genome duplication since its divergence from the last common ancestor of *T. thermophila* [14]. Comparison to the PI kinome of *S. cerevisiae* (which includes 6 PIKs) reveals a significant expansion of PIK genes in ciliates. Here, we describe in detail the members of each PIK group and discuss their functional significance and the emerging implications for the evolution of PI functions in eukaryotic cells.

## Methods

The *Tetrahymena thermophila* genome [13] was probed with human PIPKIα, PI3Kγ (PI3K Ib catalytic subunit), PI4KΙΙΙα and PI4KIIα kinase domains at NCBI using BLASTP. Additional searches included as queries yeast FAB1 and LSB6. All gene models were retrieved from the 2008 version of *T. thermophila* genome available at the Tetrahymena Genome Database (TGD Wiki, http://ciliate.org) [32]. RNA deep sequencing data from Xiong et al. (TetraFGD site, http://tfgd.ihb.ac.cn/) [33] were used to authenticate the integrity of all PIK domains identified. Some PIK gene models at TGD Wiki were not fully supported by RNA sequencing data and we used base coverage plots from the Xiong et al. study to correct the respective gene models. This resulted, amongst others, in the deletion of a RING domain in PI4K2 and PIPK2b and the deletion of a preprotein translocase and a N-terminal SecY domain in PIPK5 (for details see Table S1). One additional candidate gene, TTHERM_00637120, was eliminated since it corresponded to a MORN-motif-rich protein. A second candidate PIPK, TTHERM_00922920, which codes for a transmembrane Got1 domain-containing protein with a PIPKc domain, was found to be a mispredicted gene since the PIPKc-like domain is not expressed at all as judged by RNA sequencing [33]. *Paramecium* PIKs were subsequently identified by BLASTP searches with representative TtPIKs and retrieved from ParameciumDB (http://paramecium.cgm.cnrs-gif.fr/) [34]. Reciprocal BLASTP searches with representative PtPIKs at the non-redundant database of NCBI retrieved all identified ciliate PIKs. Predicted gene products were analyzed for domain structure at the SMART database (http://smart.embl-heidelberg.de/) and the PFAM database (http://pfam.sanger.ac.uk/). Domain boundaries and e-values for PIPKc domains have been updated using the PFAM 25.0 release and this resulted in significantly improved annotations and e-values for ciliate PIPKc domains. Putative transmembrane regions in *Tetrahymena* TtPIPK2 gene products were verified and further analyzed by ΗΜΜΤΟΡ (http://www.enzym.hu/hmmtop/) and ΗΜΗΜΜ (http://www.cbs.dtu.dk/services/TMHMM-2.0/). The PH domain in TtPI4K1 and PI3Ka domains in TtPI4K2-6 were identified by sequence alignments. For eukaryotic PIPKs used for phylogenetic analyses, genomes of representative species from alveolates, amoebozoa, excavates, choanoflagellates, chromists, metazoa, fungi and plants were searched at NCBI-BLASTP using as queries the PIPKc domains of MmPIPKIα, ScMss4, TtPIPK1a, TtPIPK2a, and TtPIPK3 or PtPIPK3a. Recovered hits were included if already annotated as PIPKs and/or if they had a PIPKc domain with a PFAM e-value<10^−18^. The locus tags, gene structure, domain boundaries and e-values of all ciliate PIKs are listed in Tables S1 and S2. Accession numbers of PIPKs from other organisms that were used for sequence alignments and phylogenetic tree construction are listed in Table S3.

The *Tetrahymena* PH cohort was retrieved at the SMART database and was further enriched by top scoring hits of a BLASTP search with the PH domain of TtPLC3 [27]. All PH-domain containing proteins were further characterized for additional domains. A ClustalW-generated cladogram was used to detect the positions and relationships of PHK genes. PHK2, 5 and 10 were found to be classified as PKB/Akt kinases by Eisen et al. [13] and the Kinome.org site (http://kinase.com/tetrahymena/).

For *Tetrahymena* PIK expression analysis, primary data were extracted from the microarray experiments of Miao et al. [35] at the TGED site (http://tged.ihb.ac.cn/) and replotted according to four culture conditions or to show relative changes during starvation or conjugation. EST data collections from Coyne et al. [36] were searched to establish expression of PIKs that were not included in the Miao et al. study. For gene network analysis of *Tetrahymena* type III PI4Ks the available networks [37] were retrieved at the TetraFGD site (http://tfgd.ihb.ac.cn/). In order to establish the validity of this approach we first searched if there are co-regulated genes in the *Tetrahymena* PIK networks that code for enzymes that may have a shared PI substrate/product relationship with each PIK. At least 13 such pairings were recovered that included either a PIK from another family (for example, TtPI4K6 vs. TtPIPK5), a PLC (for example, TtPIPK2b vs. TtPLC3), or a putative PI phosphatase. We further characterized putative PI phosphatases in the *T. thermophila* genome and found 8 synaptojanin-like genes, two of which, TtSJL4 (TTHERM_00621470) and TtSJL1 (TTHERM_00931900), were co-regulated with TtPI4K4 and TtPIPK1a, respectively. In addition, we identified a SAC family phosphatase, TtFIG4 (TTHERM_00656010), that was co-regulated with both TtPI3KIII and TtPIPK3. Lastly, a myotobularin-related phosphatase, TtMTM1 (TTHERM_00293460), was found to be co-regulated with TtPI3KIII. Co-regulated genes of the PI4K1, PI4K4 and PI4K6 networks were queried at TGD Wiki to identify annotated genes and any assigned Gene Ontology classification. In case of no annotations, relevance to a particular cellular process or metabolic activity was inferred by the presence of a human or yeast homolog. *Tetrahymena* PI4K networks were also analyzed using the functional annotation clustering module of the DAVID platform [38]. For PI4K4 and PI4K6 networks, the clusters corresponding to small GTPase mediated signal transduction (GO entry 0007264, cluster enrichment score = 1.1, p-value = 5 e^−4^) and protein kinase activity (GO entry 0004672, cluster enrichment score = 1.5, p-value = 0.004) were recovered, respectively. Details for *Tetrahymena* protein kinase and Rab gene classification were as in references 13 and 16, respectively.

Kinase catalytic domain boundaries were retrieved by the PFAM database and all amino acid alignments were performed with ClustalW or the AlignX application of the Vector NTI package. For phylogenetic analyses of ciliate PI4Ks and PIPKs, alignments were constructed and manually edited to remove large unaligned regions that contained inserts or gaps. Further elimination of positions containing gaps was performed with the complete deletion option of the MEGA version 4.0 software. The final datasets used for ciliate PI4K and PIPK tree construction contained 207 and 144 positions, respectively. Evolutionary relationships were inferred using the neighbor-joining method and the Poisson correction method for amino acid substitution and in all cases the bootstrap consensus tree inferred from 1000 or 5000 replicates is shown. For phylogenetic analysis of eukaryotic PIPKs, we additionally used the maximum likelihood method of the PhyML v3.0 program at http://mobyle.pasteur.fr and the JTT model of amino acid substitution. The final data set in this case contained 122 positions. All trees were visualized using the MEGA version 4.0 software [39].

## Results and Discussion

### Phosphatidylinositol 4-kinases (PI4Ks)

**Figure 1:**
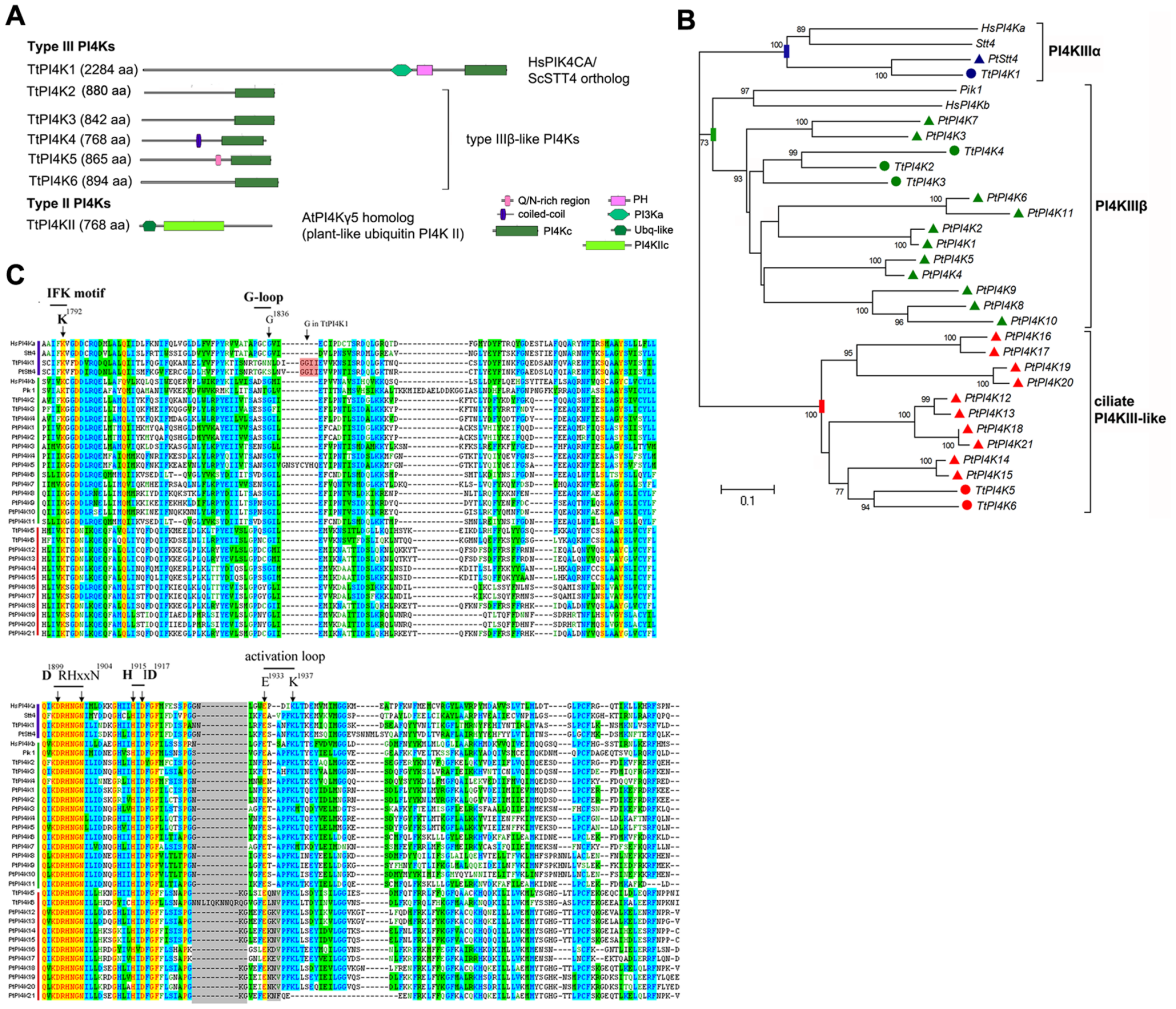
Domain structure, phylogeny and conservation of critical catalytic features of ciliate PI4Ks. A, Domain structure of *Tetrahymena* PI4Ks. The RING domain predicted in PI4K2 has been removed (see Table S1 and Methods). Domain boundaries, e-values and further details are given in Table S1. B, Unrooted neighbor-joining tree of catalytic domains from 28 ciliate, 2 yeast (Stt4, Pik1) and 2 mammalian (PI4Ka and PI4Kb) type III PI4Ks. Bootstrap values from 1000 replicates higher than 70% are indicated near the corresponding branches. Type IIIα, IIIβ and ciliate-specific III-like PI4Ks are color coded (blue, green and red, respectively). Circles and triangles represent *Tetrahymena* and *Paramecium* PI4Ks, respectively. Bar indicates number of amino acid substitutions per site. C, Sequence alignment of the catalytic kinase domains from type III ciliate, mammalian and yeast PI4Ks. The position of prominent catalytic features is indicated by arrows and numbered residues refer to human PI4KIIIα sequence [58]. All ciliate PI4Ks have conserved catalytic motifs and they bear key residues: the K^1792^ residue (IFK motif), the catalytic motifs DRH^1901^-N^1904^ and HID^1917^ and the E^1933^ and K^1937^ residues in the activation loop [58]. Significant conservation was also observed in the G-loop (G^1836^ in human PI4KIIIα). The TtPI4K1 and PtStt4 G-loop (highlighted in red) could not be properly aligned due to a unique 5aa insertion in both PI4Ks. Also, alignment of the activation loop and the E^1933^ and K^1937^ residues was distorted due to unique insertions in all type III-like ciliate PI4Ks (highlighted in grey). PtPI4K21 apparently lacks a portion of the activation loop.

We identified 7 genes that code for PI4Ks in the *T. thermophila* genome. These were further classified into 6 type III enzymes and 1 type II enzyme (Figure 1A). All type III enzymes had a C-terminal PI3K/PI4K catalytic domain but only one of them, TtPI4K1, possessed also a PI3K helical domain [1]. Nevertheless, inspection of whole sequence alignments with *Tetrahymena* PI3Ks revealed significant sequence similarity within a region of approximately 200 aa in the N-terminal half of all PI4KIII isoforms. This may indicate the presence of a cryptic PI3K helical domain in all type III PI4Ks (Table S1). An expanded set of 25 PI4K genes (22 type III and 3 type II enzymes) was also identified in *P. tetraurelia* (Table S2).

#### Ciliate type II PI4Ks

TtPI4KII possesses a N-terminal ubiquitin-like domain in addition to a PI4KII-type catalytic domain (Figure 1) and displays significant similarity to *A. thaliana* PI4Kγ5 and related genes [40]. BLAST searches in the *P. tetraurelia* genome with TtPI4KII resulted in identification of 3 genes with the same domain organization that represent *Paramecium* PI4KII enzymes (Table S2). Previous phylogenetic analyses have suggested a more close relation of plant and apicomplexa PI4KII catalytic domains when compared to animal and yeast PI4KII [7] and our results suggest that this is the case for ciliates also. PI4KII enzymes play crucial roles in Golgi morphology and vesicular trafficking in mammalian cells [1] but, currently, it is unclear whether the ubiquitin-like domain-containing PI4KII enzymes function as PtdIns 4-kinases or protein kinases. For example, Galvao et al. have shown that the ubiquitin-like domain-containing plant PI4Kγ5 and PI4Kγ7 display protein kinase activity rather than lipid kinase activity in vitro [41]. Nevertheless, TtPI4KII expression is progressively upregulated during early phases (2–10 h) of *Tetrahymena* conjugation (Figure S1C), suggesting a role in this process.

#### Ciliate type III PI4Ks

TtPI4K1 is the *Tetrahymena* PI4KIIIα/STT4 ortholog. It encodes a large protein with an extended N-terminus followed by a PI3Ka-type domain, a PH-like domain and a C-terminal PI4Kc domain (Figure 1A and Table S1). This domain structure is typical for mammalian, plant and yeast PI4K IIIα [1], [40]. Apart from TtPI4K1, the nature of the five remaining type III PI4Ks was less apparent. BLAST searches with TtPI4K2, TtPI4K3 and TtPI4K4 showed similarity to mammalian PI4KIIIβ and *Dictyostelium* PI4Ka. However, similarity was restricted solely to the PI4Kc domain, while the frequenin-binding site and homology 2-regions of mammalian and yeast PI4KΙΙΙβ [1] were absent. Phylogenetic analysis of PI4Kc domains of TtPI4K2-6 and respective domains from mammalian and yeast PI4Ks suggested that indeed TtPI4K2-4 form a separate branch of the PI4KIIIβ group. On the contrary, TtPI4K5 and TtPI4K6 appeared to be rejected from both the PI4KIIIα and PI4KIIIβ groups (Figure 1B). A similar situation occurs in *P. tetraurelia*. We identified a single STT4/PI4KIIIα ortholog, 11 homologs of TtPI4K2-4 and 10 homologs of TtPI4K5-6 (Table S2), suggesting a significant expansion of type IIIβ and type III-like PI4Ks in ciliates (Figure 1B). Close examination of putative catalytic residues in sequence alignments with mammalian and yeast PI4Ks revealed that ciliate type III PI4Ks are most likely functional PtdIns 4-kinases (Figure 1C). Analysis of publicly available microarray data [35] showed that expression of TtPI4K3 was higher compared to that of TtPI4K1, TtPI4K4 and TtPI4K6 with no significant changes during conjugation or starvation (Figure S1A–C).

#### Multiple PtdIns4P pools in *Tetrahymena*

**Figure 2:**
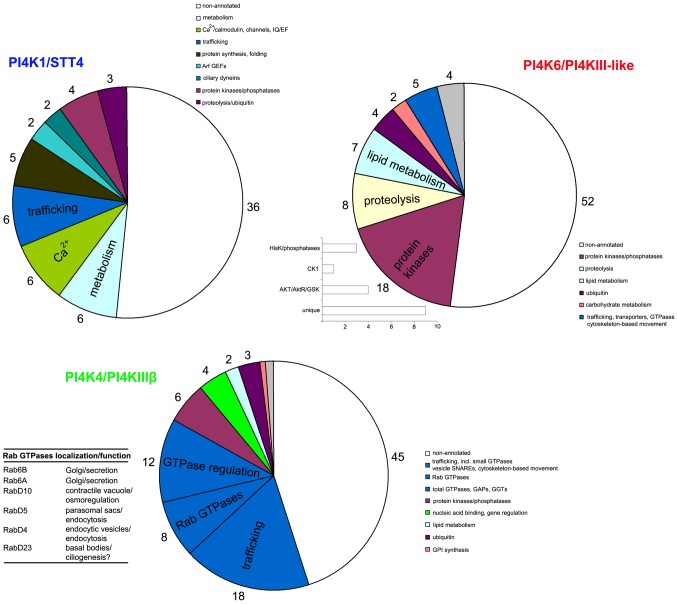
Distinct *Tetrahymena* gene networks are associated with TtPI4K1, TtPI4K4 and TtPI4K6 type III PI4Ks. Gene networks associated with the indicated TtPI4K genes were extracted from the TetraFGD site (http://tfgd.ihb.ac.cn/). The actual numbers of genes were 76, 100 and 100 for TtPI4K1, TtPI4K4, and TtPI4K6, respectively. A significant number of genes was annotated and/or characterized in terms of at least one Gene Ontology category annotation in the Tetrahymena Genome Database; percentages were 56%, 55%, and 48% for TtPI4K1, TtPI4K4 and TtPI4K6, respectively. Results are shown as pie charts indicating the number of non-annotated genes and numbers of genes involved in specific cellular processes or metabolic activities or sharing structural/functional homology. The graph in the PI4K6 panel shows the classification of TtPI4K6-associated protein kinases. The table in the PI4K4 panel shows the names, localization and possible function of TtPI4K4-associated Rabs. Two more Rab-like genes that were recovered are not included in the Bright et al. study (reference 16 in the manuscript) and they were omitted from this table.

Previous studies in *S. cerevisiae* and mammalian cells have suggested that STT4 (PI4KIIIα) and PIK (PI4KΙΙIβ) may regulate functionally distinct PtdIns4P pools [1], [42]. In *Tetrahymena*, PtdIns4P has been suggested to be involved in certain signaling and vesicular trafficking pathways, namely, osmotic stress and secretion [26]. Furthermore, the co-expression of the 6 different type III PI4Ks suggests a broader capacity for regulation of distinct PtdIns4P pools. In order to provide additional support for this, we searched for the *T. thermophila* gene networks [37] associated with specific PI4KIII genes. We analyzed data for TtPI4K1, TtPI4K4 and TtPI4K6, available at the TetraFGD (http://tfgd.ihb.ac.cn/). The validity of this approach was assessed as described in Methods. A significant number of co-regulated genes was annotated in the Tetrahymena Genome Database and this permitted a functional analysis. As shown in Figure 2, PI4K1, PI4K4 and PI4K6 were characterized by distinct networks associated with different cellular processes or functions. While PI4K1 was associated with genes involved in metabolism, Ca^2+^ regulation and trafficking, the ciliate-specific PI4KIII-like PI4K6 was primarily associated with protein kinases and phosphatases involved in protein phosphorylation. The majority of these protein kinases appear to belong to *Tetrahymena*-specific kinase families and groups (termed “unique” in the inset of the PI4K6 panel in Figure 2). Analysis, of the PI4KIIIβ/PI4K4 gene network revealed an enrichment of genes involved in membrane trafficking. Amongst this network at least 8 Rab GTPases, 2 Arf-like GTPases and genes involved in GTPase regulation and membrane association were recovered (Figure 2). A recent detailed functional study showed that most of the specific Rab genes recovered in the PI4K4 network are primarily associated with Golgi secretory pathway and endocytosis [16]. This points to a putative function of TtPI4K4-generated PtdIns4P in the regulation of these specific trafficking pathways. Furthermore, mammalian PI4KIIIβ and yeast PIK1 have been characterized as key enzymes in regulating Golgi function and morphology and both interact with Rab GTPases (at least Rab11) via their homology2 region [1]. The data for TtPI4K4, thus, may also suggest a close evolutionary conservation of the PI4KIIIβ-Rab-Golgi interaction despite clear topological and morphological differences in Golgi networks of mammalian and *Tetrahymena* cells [16].

### Phosphoinositide 3-kinases (PI3Ks)

**Figure 3:**
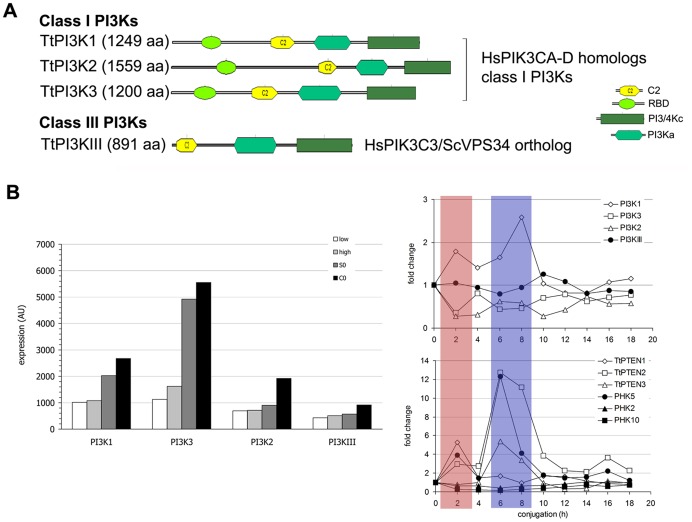
Domain structure, classification and expression patterns of *Tetrahymena* PI3Ks. A, Domain structure and classification of *Tetrahymena* PI3Ks. For domain boundaries, e-values and further details see Table S1. B, Left panel: Expression data for the TtPI3K genes were extracted from the TGED site (http://tged.ihb.ac.cn/) and replotted in order to compare the expression at four different conditions: low and high cell density during growth, start of starvation (S0) and start of conjugation (C0). AU, arbitrary units. B, Right panel: Expression data for TtPI3Ks, TtPTENs and TtPHK2, 5 and 10 during conjugation were normalized relative to controls (time 0) and are plotted as fold changes. The overlapping patterns of upregulation of TtPI3K1, TtPHK5 and TtPTEN1-3 are highlighted by boxes.

An early search for putative PI3K genes in *Tetrahymena*, before the completion and annotation of its genome, had revealed 4 putative genes which were tentatively characterized as 3 class I and a single class III PI3K [25]. The 3 *Tetrahymena* class I PI3Ks (TtPI3K1-3) have the typical class I domain organization consisting of RBD, C2, PI3Ka and C-terminal PI3Kc domains. TtPI3KIII, the *Tetrahymena* PI3K class III/VPS34 ortholog, responsible for the synthesis of PtdIns3P in *Tetrahymena* [25], codes for a shorter protein lacking the RBD domain (Figure 3A and Table S1). We were unable to identify any regulatory subunits for TtPI3K1-3, but the regulatory subunit of PI3KIII/VPS34 orthologs, known as VPS15 in *S. cerevisae*, is encoded by TTHERM_00543659. Alignment of the catalytic domain with class I/III enzymes from mammals revealed conservation of all catalytically important residues in the PI3Kc domain [25].

#### *Tetrahymena* PI3KI-PDK1-PTEN-PKB signaling axis

The presence of a set of class I PI3Ks in *Tetrahymena* suggests that these enzymes, previously inferred as metazoa and amoebozoa-specific [2], [6], are more widespread in the eukaryotic kingdom. In support of this, we identified 5 PI3K class I genes in *Paramecium* (Table S2).

Class I PI3Ks are master-regulators of cell growth, metabolism and chemotaxis in mammals, the model organisms *D. melanogaster* and *C. elegans* and the unicellular slime mold *D. discoideum* [2], [6]. In these organisms, production and signaling by PtdIns(3,4,5)P_3_, the product of PI3K I, depends on downstream mechanisms that relay or terminate PI3K activity, such as PH domain-containing PDK1 and PKB/Akt protein kinases or PTEN phosphatase, respectively [2]. We analyzed a cohort of PH-like domains in *Tetrahymena* gene products and identified 12 PH-domain-containing kinases (named TtPHK1-12) (Figure S2A). TtPHK12 (TTHERM_00188930) is identical to the single PDK1 homolog that has been identified in the *T. thermophila* genome [13]. TtPDK1/PHK12 has a C-terminal PH domain and searching with the HMMER tool revealed a strong similarity to the PH domain of human PDK1 (e-value = 4.6×e^−5^). Furthermore, sequence alignments with PH domains from PDK1 orthologs from several species revealed that the *Tetrahymena* PDK1-PH domain retains key interactions with the D3- and D4-phosphates of PtdIns(3,4,5)P_3_ (Figure S2B) [43]. Three additional PH domains (those in TtPHK2, 5 and 10) cluster together and are loosely associated with the PH domain of TtPDK1 in indicative ClustalW-generated cladograms (Figure S2A). We tested the classification of all PHK genes in the original tentative characterization of the *Tetrahymena* kinome [13], and found that TtPHK2, 5 and 10 have been designated as putative AGC/Akt kinases. TtPHK2 and TtPHK10 have additional ankyrin motifs N-terminal to the PH domain and only TtPHK5 bears the typical domain architecture of PKB/Akt (Figure S2C). Furthermore, we identified 3 catalytically functional PTEN paralogs in the *T. thermophila* genome (TtPTEN1-3; Figure S2D). Similar sets of PKB/Akt-like kinases and PTEN paralogs were also identified in the *P. tetraurelia* genome (see legend in Figure S2). These results suggest that PTEN-dependent inhibition of PI3K I signaling, via dephosphorylation of PtdIns(3,4,5)P_3_, is likely to be functional in these organisms as well.

#### Evidence for PI3K class I-specific signaling during *Tetrahymena* conjugation

All TtPI3Ks are expressed under various culture conditions as indicated by EST database searches and microarray data [35], [36]. TtPI3KIII, in particular, was stably expressed throughout vegetative growth and conjugation and only slightly increased upon starvation, in agreement with its suggested house-keeping role in constitutive trafficking [25]. The expression pattern of TtPI3K1-3 was more complex including an abrupt upregulation (2-3-fold) upon initiation of starvation and conjugation (Figure 3B). These changes, however, are difficult to interpret since they occur upon centrifugation and resuspension of cells in starvation medium, as noted previously [16]. TtPI3K1-3 genes are likely to be involved in *Tetrahymena* phagocytosis/autophagy and chemotaxis pathways (our unpublished data). Yet, an unexpected putative role for a class I PI3K was revealed by analysis of expression patterns during conjugation. PI3K1 expression peaked at 2 h (1.8-fold) and 8h (2.5-fold), while PI3K2-3 expression remained decreased throughout conjugation (Figure 3B). We next analyzed the expression patterns of PTEN and PKB/Akt paralogs during conjugation. We found that TtPTEN1 peaked 5-fold at 2 h while TtPTEN2 and TtPTEN3 increased approximately 12- and 5-fold, respectively, at 6–8h of conjugation (Figure 3B). Also, TtPHK5 expression peaked at 2 h (4-fold) and at 6–8 h (11-fold) of conjugation (Figure 3B). These results suggest that key components of a putative PI3K-PDK1-PTEN-PKB axis in *Tetrahymena* exhibit partially overlapping peaks of expression in conjugating cells. Considering the well established timing of *Tetrahymena* conjugation, the 2 h time point corresponds to cell pairing and the 6–8 h time points corresponds roughly to new macronucleus development [35]. The specific upregulation of expression during the 6–8 h time points, in particular, directly corroborates previous pharmacological inhibition studies which have suggested that PI3K signaling may regulate programmed parental macronuclear degradation during conjugation [44], [45]. Collectively, these results point to a unique role of PI3K I signaling in *Tetrahymena*.

### Phosphatidylinositol phosphate kinases (PIPKs)

**Figure 4:**
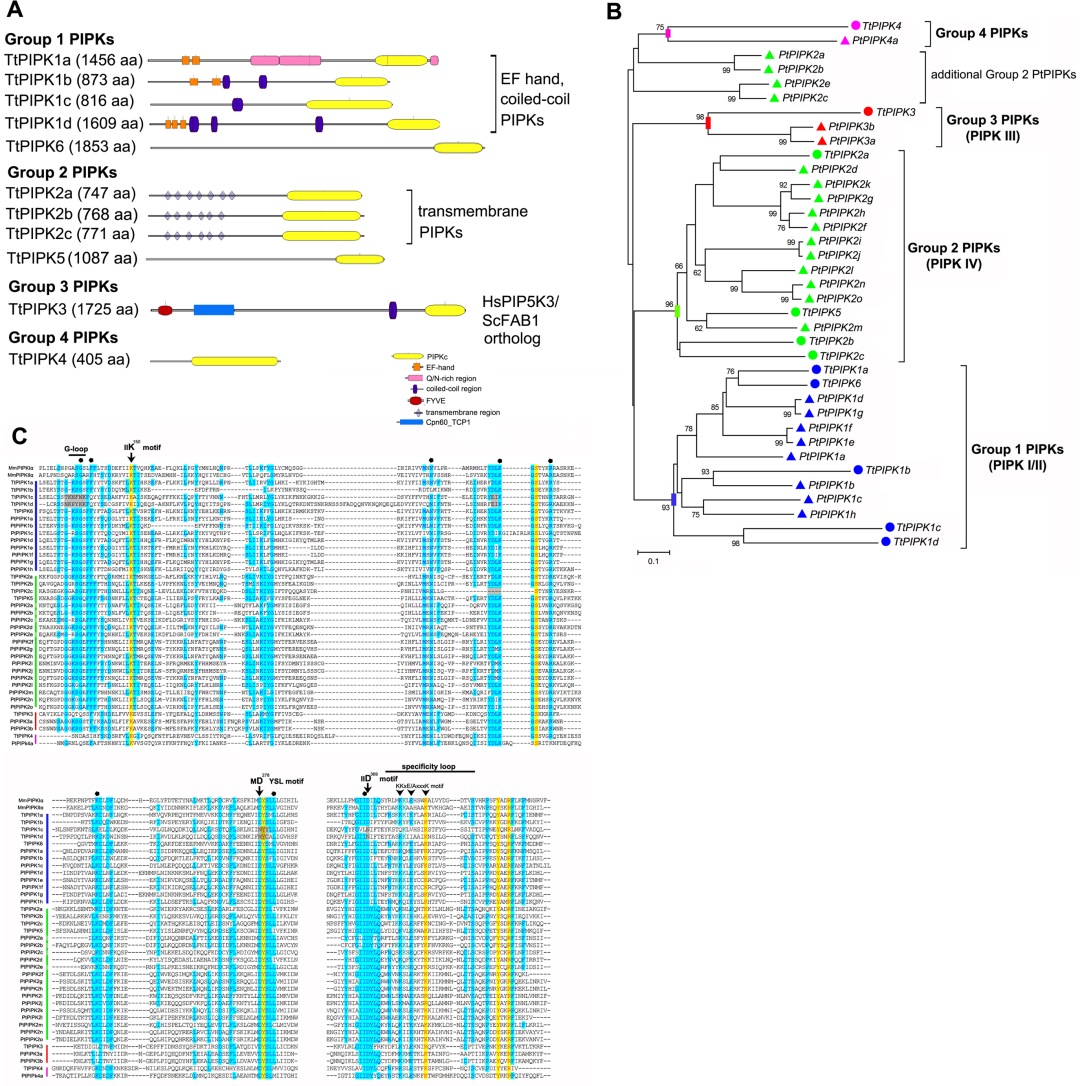
Domain structure, phylogeny and conservation of critical catalytic features of ciliate PIPKs. A, Domain structure of *Tetrahymena* PIPKs. The RING domain predicted in PIPK2b and transmembrane and SecY domains in PIPK5 have been removed (see Table S1 and Methods). Domain boundaries, e-values and further details are given in Table S1. B, Unrooted neighbor-joining tree of catalytic domains from 37 ciliate PIPKs. Bootstrap values from 5000 replicates higher than 60% are indicated near the corresponding branches. Group 1, 2, 3 and 4 PIPKs are color coded (blue, green, red and purple respectively). Circles and triangles represent *Tetrahymena* and *Paramecium* PIPKs, respectively. Bar indicates number of amino acid substitutions per site. Phylogenetic relationships of ciliate group 2 PIPK genes were less resolved with less nodes supported by high bootstrap values. In *Paramecium*, 4 additional group 3 PIPKs that are organized in 2 pairs of paralogs (PtPIPK3c,d and PtPIPK3e,f) and they are most related to TtPIPK3 are not shown. C, Sequence alignment of the catalytic kinase domains from ciliate PIPKs and mammalian PIPKIα and PIPKIIα. The position of prominent catalytic features is indicated by arrows and arrowheads and numbered residues refer to the mouse PIPKIIβ structure described in reference 46. Polygons indicate residues that interact with ATP or the phosphoinositide substrate (PtdIns5P) in the crystal structure of PIPKIIβ and they are conserved in both type I and II PIPKs [46]. The variable inserts between the MDYSL and IID motifs present in all PIPKs have been omitted. The residues K^150^, D^278^ and D^369^, essential for catalytic activity, are conserved in all but 2 *Tetrahymena* PIPKs (highlighted in grey; see text and Figure S4 for details). The DLKGS motif in TtPIPK2c (highlighted in grey) has been reconstituted from RNA sequencing data (Table S1). The position of the KKxE/AxxxK motif in the specificity loop is indicated by a bar; further K residues that may contribute are highlighted by light blue and most ciliate PIPK1, but not PIPK2, genes confront to the consensus KK motif. Note that in all but 2 ciliate PIPKs the +2 position (E/A residues) in the specificity loop is occupied by a Glu residue as in all PtdInsP 5-kinases.

Our search for proteins with a PIPK domain in the *T. thermophila* genome resulted in the identification of 11 putative PIPK genes. Based on PIPKc domain phylogenetic analysis and overall domain structure, *Tetrahymena* PIPKs are classified into four groups (TtPIPK1-4) (Figure 4). A total of 30 PIPKs that were identified in the genome of *P. tetraurelia* were also classified in to these 4 groups (PtPIPK1-4) suggesting a strong conservation of ciliate PIPKs (Figure 4B and Table S2).

#### The ciliate type IV transmembrane PIPKs

Analysis of the domain structure of TtPIPK2a-c showed that they are transmembrane proteins (Figure 4A). TtPIPK2a possesses a signal peptide region followed by 7 well-characterized transmembrane regions assuming the overall structure of a 7TM protein with a cytosolic PIPK domain. Topological analysis of TtPIPK2b and 2c showed the presence of 6 transmembrane regions that resulted in a luminal orientation of their PIPKc domains. In both proteins, however, a seventh transmembrane region that is predicted with below threshold probabilities may switch this orientation (not shown). Initial assignment of PIPK5 revealed a transmembrane preprotein translocase with a N-terminal SecY domain. However, close inspection of RNA sequencing data revealed that PIPK5 codes for a shorter protein lacking transmembrane motifs and the SecY domain (see Methods and Table S1). Nevertheless, our phylogenetic analysis suggested that PIPK5 is homologous to group 2 PIPKs (Figure 4B). Alignment of PIPKc domains revealed conservation of the IIK, MDYSL and IID catalytic motifs in all group 2 PIPKs, but PIPK2a and PIPK5 had a QK motif instead of the KK motif in the activation loop (Figure 4C). This, according to the crystal structure and mutagenesis studies of PIPKIIβ, is suggestive of a reduced substrate affinity [3], [46].

**Figure 5:**
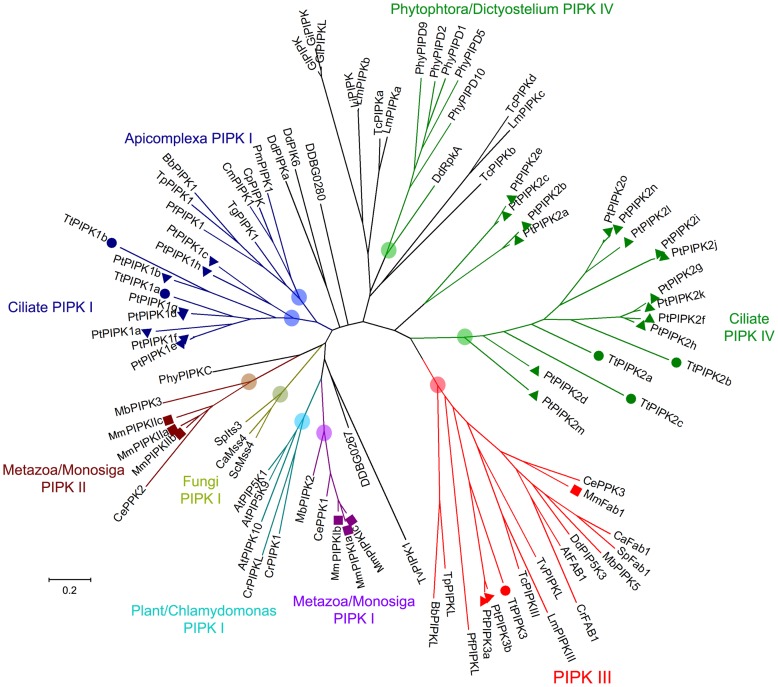
Phylogenetic analysis of PIPKs reveals evolutionary conservation of PIPKIII/FAB1 orthologs in most unicellular organisms. Unrooted maximum likelihood tree of catalytic domains from 92 eukaryotic PIPKs. Ciliate and apicomplexa group 1 PIPKs (reassigned as type I PIPKs due to the characterization of PfPIPK/NCS [51]), ciliate and *Dictyostelium*/*Phytopthora* type IV PIPKs, type III PIPKs, metazoa/*Monosiga* type I and II PIPKs as well as fungi and plant/*Chlamydomonas* PIPKs are color-coded as indicated. Circles indicate *Tetrahymena* PIPKs, triangles *Paramecium* PIPKs and squares mammalian PIPKs. Bar indicates number of amino acid substitutions per site. Nodes that are supported by bootstrap analysis (1000 replicates, >69%) and neighbor-joining trees are highlighted according to the color code of each group. For PIPK repertoires of selected organisms, accession numbers and further details see Methods and Table S3.

It is clear that *Tetrahymena* PIPK2a-c genes are members of a recently proposed type IV transmembrane PIPK group which also includes PIPKs from *D. discoideum* (RpkA) and *Phytopthora* (12 genes) [47], [48]. We identified 15 additional members of this group in *P. tetraurelia* (Figure 4B and Table S2). Furthermore, we found a similar PIPK in the choanoflagellate *Monosiga brevicollis* genome (MbPIPK4, Table S3). This, suggests a more widespread occurrence than previously assumed [47]. The unique 7TM-PIPK structure of most type IV PIPKs points to a common phylogenetic origin. Indeed, our phylogenetic analyses suggested a common origin for most ciliate genes including TtPIPK2a-c, TtPIPK5 and 11 *Paramecium* PIPK2 genes (Figure 4B and Figure 5). Nevertheless, most phylogenetic tree construction models failed to place all type IV PIPKs, or, at least *Phytophtora* and ciliate genes, into a common phylogenetic group. Furthermore, a subgroup of *Paramecium* PIPK2s (PtPIPK2a,b,c,e genes) appeared to be rejected from the ciliate PIPK2 clade (Figure 4B and Figure 5). In addition, the PIPK domain of MbPIPK4 gene was the most divergent and was never confidently grouped with DdRpkA, ciliate or *Phytopthora* type IV PIPKs (data not shown). Thus, in the absence of solid phylogenetic support, we propose that type IV PIPKs may have evolved independently via convergent evolution in certain eukaryotic lineages.

To date, there are no in vitro or in vivo data for catalytic function and substrate specificity of type IV PIPKs. Yet, loss of RpkA in *Dictyostelium* results in reduced ^32^P-labelling of PtdInsP and PtdInsP_2_, suggesting that it regulates phosphoinositide synthesis [49]. Overall conservation of catalytically important residues in type IV PIPK catalytic domains and comparison of their activation loop regions to those of PIPKI-III suggested that they are most likely PtdInsP-5 kinases (Figure 4C). Type IV PIPKs are permanently associated with membranes in contrast to type I/II PIPKs which can interact transiently with plasma membrane [46], [50]. This different mode of membrane association suggests that different mechanisms of regulation of PIPK activity may be employed for type IV PIPKs compared to type I/II PIPKs. For *Tetrahymena*, in particular, this imposes a significant issue since PIPK2a is the most highly expressed gene throughout growth, starvation and conjugation, reaching 15-fold higher expression compared to other PIPK (Figure S5) or even PI4K and PI3K genes (Figure S1A and Figure 3B).

#### Ciliate PIPK1 genes are members of an alveolate-specific PIPK I/II group

Analysis of TtPIPK1a-d and TtPIPK6 showed that they contain variable numbers of EFh motifs and/or coiled-coil regions in their N-termini. A similar PIPK, named PfPIP5K/NCS, has been characterized in the apicomplexan parasite *Plasmodium falciparum*. PfPIPK/NCS displays PtdIns4P 5-kinase activity and is activated by the small GTPase Arf1 but not by phosphatidic acid [51]. We were able to identify relatives of this type of PIPKs in all apicomplexa sequenced genomes and also in *P. tetraurelia* (8 genes, Figure 4B and Figure 5, Tables S2–S3). Apparently, as suggested also by Brown and Auger [7], this group of PIPKs may constitute a phylogenetically distinct, alveolate-specific, subfamily of type I PIPKs (Figure 5). The EFh motifs of PfPIPK/NCS bear some similarity to neuronal calcium sensor protein-1 (NCS-1)/frequenin and it has been proposed that this may allow modulation of PtdIns(4,5)P_2_ synthesis in response to changes in Ca^2+^ [51]. Although this has not been directly tested, we have identified a conspicuous relationship between Ca^2+^ and PtdInsP_2_ levels in *Tetrahymena*. For example, we have found that total PtdInsP_2_ levels are increased by treatment with the Ca^2+^ ionophore A23187 in an EGTA-sensitive manner and, also, that the addition of Ca^2+^ in deciliation experiments results in abrupt increase of PtdIns(4,5)P_2_ in ciliary membranes (our unpublished data).

Analysis of the expression patterns of TtPIPK1 genes revealed that most of them correspond to low abundance transcripts with TtPIPK1a being the most highly expressed gene (Figure S3A). TtPIPK1a has no substitutions of catalytically important residues, suggesting an efficient catalytic activity compared to TtPIPK2a (Figure 4C). Furthermore, TtPIPK1a expression is transiently upregulated during early stages of conjugation (Figure S3B). These stages correspond to formation and stabilization of cellular pairs and chromosome segregation in the micronucleus, and possibly point to a specific role for TtPIPK1a-generated PtdIns(4,5)P_2_ during conjugation.

#### Identification of pseudo-PIPKs in protists

Detailed analysis of sequence alignments of ciliate PIPKs revealed that TtPIPK1c and d genes code for apparently inactive enzymes. Their PIPKc domains, despite scoring significant e-values (e^−53^ and e^−45^) in the PFAM database, have Asn instead of Asp residues in the MDYSL and IID catalytic motifs [46], [50] and bear a degraded G-loop (Figure 4C). According to our knowledge this is the first identification of pseudo-PIPKs in analogy to protein pseudo-kinases [8], [10], [11]. We also identified a limited number of additional pseudo-PIPKs in other protists (Figure S4 and Table S3), suggesting that it is not a *Tetrahymena* specific trait. Interestingly, analysis of the activation loop sequences of TtPIPK1c and TtPIPK1d showed that the unique Glu residue at the +2 position after the KK motif, that is conserved in all type I PIPKs and determines specificity for PtdIns4P [3], is replaced by the aliphatic amino acids Val and Ile, respectively (Figure 4C and Figure S4 in more detail). This is reminiscent of the type II PIPK activation loop which includes an Ala residue in this position, crucial for recognition of PtdIns5P as a substrate [3]. Putative pseudo-PIPKs from other protists also lack either the catalytic Lys or Asp residues in the IIK, MDYSL, IID motifs. Furthermore, they almost all bear degraded activation loops that have substitutions of this unique +2 Glu residue and lack critical Lys residues (Figure S4). Alignment of 100 sequences of non-metazoan PIPKs revealed 12 sequences that had substitutions of this unique +2 Glu residue. Most of them were pseudo-PIPKs while the rest were apparently active PIPKs bearing a type II activation loop (Ala instead of Glu) (Figure S4). According to our knowledge, this is the first indication of the possible presence of active type II-like PIPKs in unicellular eukaryotes. This diverse group of type II-like and pseudo PIPKs included phylogenetically unrelated proteins with PIPKc domains scoring a wide range of e-values in the PFAM database (from e^−18^ for *D. discoideum* PI4P5Kb, to e^−62^ for *L. braziliensis* PIPK; Figure S4). The nature of these pseudo-PIPKs and type II-like PIPKs is entirely unknown, but they may regulate active PIPKs by heterodimer formation [52], [53]. Pseudo-PIPKs, in particular, may also utilize different catalytic mechanisms in a manner analogous to protein pseudo-kinases [10].

#### Ciliate PIPK3 genes are the PIPKIII/FAB1 orthologs

TtPIPK3 codes for a large protein with extended N-terminus bearing the typical FYVE and TCP1 domains of PIPKIII kinases (Figure 4A). Furthermore, alignment of the TtPIPK3 FYVE domain with the FYVE domains of *S. cerevisiae* FAB1 and *M. musculus* PIKfyve showed conservation of critical residues that are crucial for complexing Zn^2+^ and PtdIns3P binding (data not shown). PIPKIII orthologs share also similatity in a central region of unknown function, the PIPKIII-unique domain [54]. We were unable to detect substantial similarity of TtPIPK3 to the most conserved parts of the consensus sequence of the PIPKIII-unique domain derived from fungi, metazoan and plants [54]. In *P. tetraurelia* we found 6 genes, PtPIPK3a-f, that code for type III PIPKs all bearing the N-terminal FYVE and TCP1 domains (Table S2). These PtPIPK3 genes are organized in 3 pairs of paralogs, one of which, PtPIPK3a and 3b, is shown in Figure 4B . We extended our phylogenetic analysis of PIPKc domains to include representative species across major taxonomic groups and this revealed clear PIPKIII/FAB1 orthologs in most organisms analyzed. This universal PIPKIII/FAB1 group was supported by reasonable bootstrap statistical confidence in both neighbor-joining and maximum likelihood trees (Figure 5). Furthermore, putative PIPKIII/FAB1 orthologs from *Babesia bovis*, *Theileria parva*, *Plasmodium falciparum*, *Trichomonas vaginalis* and *Trypanosoma cruzi* (TpPIPKL, BbPIPKL, PfPIPKL TvPIPKL and TcPIPKIIII, respectively, Figure 5) lack FYVE domains but they retain phylogenetically related PIPK type III catalytic domains. This perhaps suggests the early evolutionary commitment of type III PIPKc domains to production of PtdIns(3,5)P_2_ [54]. On the contrary, the phylogenomics of type I/II PIPKc domains were much less resolved (Figure 5) [7] suggesting different evolutionary constraints for production of PtdIns(4,5)P_2_ in eukaryotic cells.

In contrast to ciliate PIPK3 genes, TtPIPK4 and PtPIPK4a (Figure 4A,B) have no apparent homologs in other eukaryotic genomes. Their PIPKc domains appear to be highly divergent (with e-values e^−40^ and 3^−38^, respectively, Table S1) but they retain apparently functional IIK, MDYSL and IID catalytic motifs and activation loops as well as most ATP/substrate interacting residues (Figure 4C).

## Conclusions

Complexity of PI metabolism in mammals constitutes an evolutionary aspect of phosphoinositide biology, yet there is limited and scarce information on the PIK repertoire in the vast majority of unicellular eukaryotes. The present study aimed at providing a comprehensive analysis of PIK genes in two free-living ciliates, *Tetrahymena thermophila* and *Paramecium tetraurelia*. We found that both these organisms have a set of class I PI3Ks, a single PH-domain-containing PDK1 gene, and expansion of PTEN paralogs that suggest a functional PI3K class I-signaling pathway. Furthermore, analysis of expression patterns indicated a role for PI3K class I signaling during macronuclear differentiation in *Tetrahymena*. Class I PI3Ks have been also detected in other protists occupying distant branches of the eukaryotic evolution tree (e.g. *Naegleria gruberi*, *Trypanosoma cruzi*, *Leishmania*, *Phytophthora*, *Giardia intestinalis*) [7], [48], [55]–[57]. Thus, it would appear that class I PI3Ks may actually represent ancestral eukaryotic kinases that were specifically lost from some eukaryotic lineages including parasitic apicomplexa, fungi and plants. Yet, according to our knowledge, definite evidence for production of PtdIns(3,4,5)P_3_ in any of those protists is still lacking. PtdIns4P, the major monophosphoinositide in eukaryotes, has multiple roles as precursor of PtdIns(4,5)P_2_ and as regulator of vesicle budding and trafficking [1], [6]. We found that both ciliates exhibit a striking expansion of PI4KIII genes, including a new class of PI4KIII-like proteins. Furthermore, gene expression data and network analysis in *Tetrahymena* indicated non-overlapping patterns of functions for PI4KIIIα, PI4KIIIβ and PI4KIII-like genes. The expansion of PI4KIII genes is reminiscent of the expansions of other gene families associated with membrane trafficking seen in both *Tetrahymena* and *Paramecium* [13], [16]–[18]. Thus, it is conceivable that ciliates have increasingly elaborated on PtdIns4P synthesis perhaps due to evolutionary constraints imposed by their life style and the necessity to regulate multiple PtdIns4P pools at their extremely complex endomembrane system [16]–[18]. PIPKs are responsible for production of the bisphosphoinositides PtdIns(4,5)P_2_ and PtdIns(3,5)P_2_ in eukaryotic organisms [6], [7]. PtdIns(4,5)P_2_, in particular, has been assigned multiple roles, being both the precursor in prominent signaling pathways and a critical regulator of exocytosis/endocytosis and actin cytoskeleton remodeling [6]. *Tetrahymena* and *Paramecium* appear to be unique in having 4 distinct types of PIPKs. PIPK3 genes are the PIPKIII/Fab1 orthologs and we were able to show that a universal eukaryotic type III PIPK group is reasonably resolved (Figure 5). This clearly indicates a solid evolutionary commitment of a subset of PIPKc domains to PtdIns(3,5)P_2_ synthesis from PtdIns3P. PIPK1 genes may be regarded as relatives of the type I/II core unicellular PIPK [6], [7], and, most likely, responsible for the canonical synthesis of PtdIns(4,5)P_2_ from PtdIns4P in these organisms [51]. Yet, the presence of PIPK2 genes (the ciliate version of type IV PIPKs), PIPK4 genes, and novel pseudo-PIPKs and type II-like PIPKs (at least in *Tetrahymena* and some other protists) suggest further elaboration on biosynthetic pathways of PtdIns(4,5)P_2_ (and perhaps other PtdInsP_2_ isomers) in unicellular eukaryotic organisms. Overall, our results provide a solid framework for future investigation of the PIK roles in phosphoinositide metabolism and cellular functions in ciliates. Furthermore, they suggest the possibility that novel functions and novel regulatory pathways of phosphoinositides may be more widespread than previously thought in the vast majority of eukaryotes.

## Supporting Information

Figure S1 **Expression patterns of** ***Tetrahymena*** **PI4K genes.** A, Expression data for the indicated PI4K genes were extracted from the TGED site (http://tged.ihb.ac.cn/) and replotted in order to compare the expression at four different conditions: low and high cell density during growth, start of starvation (S0) and start of conjugation (C0). AU, arbitrary units. Expression data for PI4K2 and PI4K5 were unavailable, but both genes are expressed with at least one EST clone detected during starvation (reference 36 in the manuscript). B and C, Expression data during starvation and conjugation were normalized relative to controls (time 0) and are plotted as fold changes. Note the striking and gradual upregulation of TtPI4KII during conjugation reaching a peak (9-fold) at 8 h and the sharp decrease and stabilization after 10 h.
(TIF)

Figure S2 **Components of a PI3K class I-associated PDK1-PKB/Akt-PTEN pathway are present in** ***Tetrahymena*** **.** A, ClustalW-generated cladogram of a cohort of PH domains in *T. thermophila* gene products retrieved from the SMART database. The two grey boxes highlight two groups of PH-containing protein kinases (PHKs; these were numbered arbitrarily and their names are shown next to the respective locus tag). Group B consists of PHK2, PHK10, PHK5 and PDK1/PHK12 PH domains. Additional PH domains from other gene products, some of which are likely to be involved in various aspects of lipid/PI metabolism, are also indicated: OPR1,2 are oxysterol-binding protein (OSBP)-related proteins; PLD1a,b are phospholipases D; PLC3 is the inactive *Tetrahymena* PI-specific phospholipase C PRIP-like protein (reference 27 in the manuscript) and b indicates hits from BLAST analysis with the PLC3 PH domain that were included in the cohort; GRP1 is identical to the TtTST1 gene (a TBC-Sec7 family Arf-GEF) related to the previously described PH domain-containing TtGEF1 gene product (a GBF/BIG family Arf-GEF) (Bell et al., 2009, Cell Motil Cytoskeleton 66∶483–499; Awan et al., 2009, PLoS One 4(3):e4873); PHC1,2 are PH-containing adenylyl/guanylyl cyclases. PH-domain containing proteins with putative transmembrane regions, coiled-coil regions or low-scoring domains detected by SMART or PFAM databases are not indicated. B, Sequence alignments of PH domains from PDK1 orthologs reveals conservation of critical PtdIns(3,4,5)P_3_ D3/D4-phosphate interacting residues. *Tetrahymena* (and *Paramecium*) PDK1-PH domains retain key interactions with the D3-phosphate (residues K465 R474, K495; numbering refers to hPDK1 residues) and D4-phosphate (residues K465, Y486, R521) but not the D5-phosphate of PtdIns(3,4,5)P_3_ (residue K467 is replaced by V in TtPDK1) (reference 43 in the manuscript). The gene locus tag of PtPDK1 is GSPATT00036680001. PDK1 orthologs from *Homo sapiens* (Hs), *Mus musculus* (Mm), *Danio rerio* (Dr), *Drosophila melanogaster* (Dm) and *Dictyostelium discoideum* (Dd) were retrieved from the respective genome databases. C, Domain structure of *Tetrahymena* PKB/Akt-like kinases (PHK2, 5 and 10) and PDK1. The *P. tetraurelia* genome contains 4 homologous Akt-like kinases with the same ankyrin-PH domain structure (GSPATP00004764001, GSPATP00003167001, GSPATP00003075001, GSPATP00020061001). D, Domain structure and catalytic motif of *Tetrahymena* PTEN paralogs. PTP-DSPc, protein tyrosine phosphatase-dual specificity phosphatase catalytic domain; PTEN_C2, C2-homology domain of PTEN. Alignment of TtPTENs and HsPTEN PTP-DSPc domains indicates the conservation of the CX_5_R catalytic motif and the Cys residue (in red) essential for PTEN activity. Corresponding e-values for the PTP-DSPc and PTEN_C2 domains were: HsPTEN (e^−4^/e^−50^), TtPTEN1 (e^−2^/e^−32^), TtPTEN2 (e^−3^/e^−28^) and TtPTEN3 (e^−3^/e^−20^). At least ten *Paramecium tetraurelia* PTEN paralogs share significant similarity to TtPTEN1 (e-values <10^−66^) and bear a conserved CX_5_R catalytic motif.
(TIF)

Figure S3 **Expression patterns of** ***Tetrahymena*** **PIPK genes.** A, Expression data for the indicated PIPK genes were extracted from the TGED site (http://tged.ihb.ac.cn/) and replotted in order to compare the expression at four different conditions: low and high cell density during growth, start of starvation (S0) and start of conjugation (C0). AU, arbitrary units. Note that TtPIPK1b,c, TtPIPK2b,c and TtPIPK4 are expressed at low levels during vegetative growth. B, Expression data during conjugation were normalized relative to controls (time 0) and are plotted as fold changes. The unique and transient upregulation of TtPIPK1a during 2–4 h of conjugation (3-4-fold) is highlighted by a box.
(TIF)

Figure S4 **Occurrence of pseudo-PIPKs in protists.** Substitution of key residues in the G-loop, IIK, MDYSL, IID motifs and/or the activation loop of the indicated pseudo-PIPKs is highlighted by red boxes. TtPIPK1c and TtPIPK1d are indicated by arrowheads. Four apparently active protist PIPKs that bear a type II activation loop (A residue in the +2 position) and representative mammalian type I and II PIPKs are also included in the alignment for comparison. Of the four protist type II PIPKs only the *Monosiga brevicolis* MbPIPK3 is phylogenetically related to metazoa PIPKII (see Figure 5). The accession numbers of PIPKs are listed in Table S3.
(TIF)

Table S1 ***Tetrahymena*** **PIKs.**
(DOC)

Table S2 ***Paramecium*** **PIKs.**
(DOC)

Table S3 **Eukaryotic PIPKs used for alignments and phylogenetic trees.**
(DOC)
