# Supplementary figures and images for: Genome-Wide Analysis of the Phosphoinositide Kinome from Two Ciliates Reveals Novel Evolutionary Links for Phosphoinositide Kinases in Eukaryotic Cells
Source: PLoS One. 2013 Nov 11;8(11):e78848. doi: 10.1371/journal.pone.0078848 (PMC3823935; doi:10.1371/journal.pone.0078848)

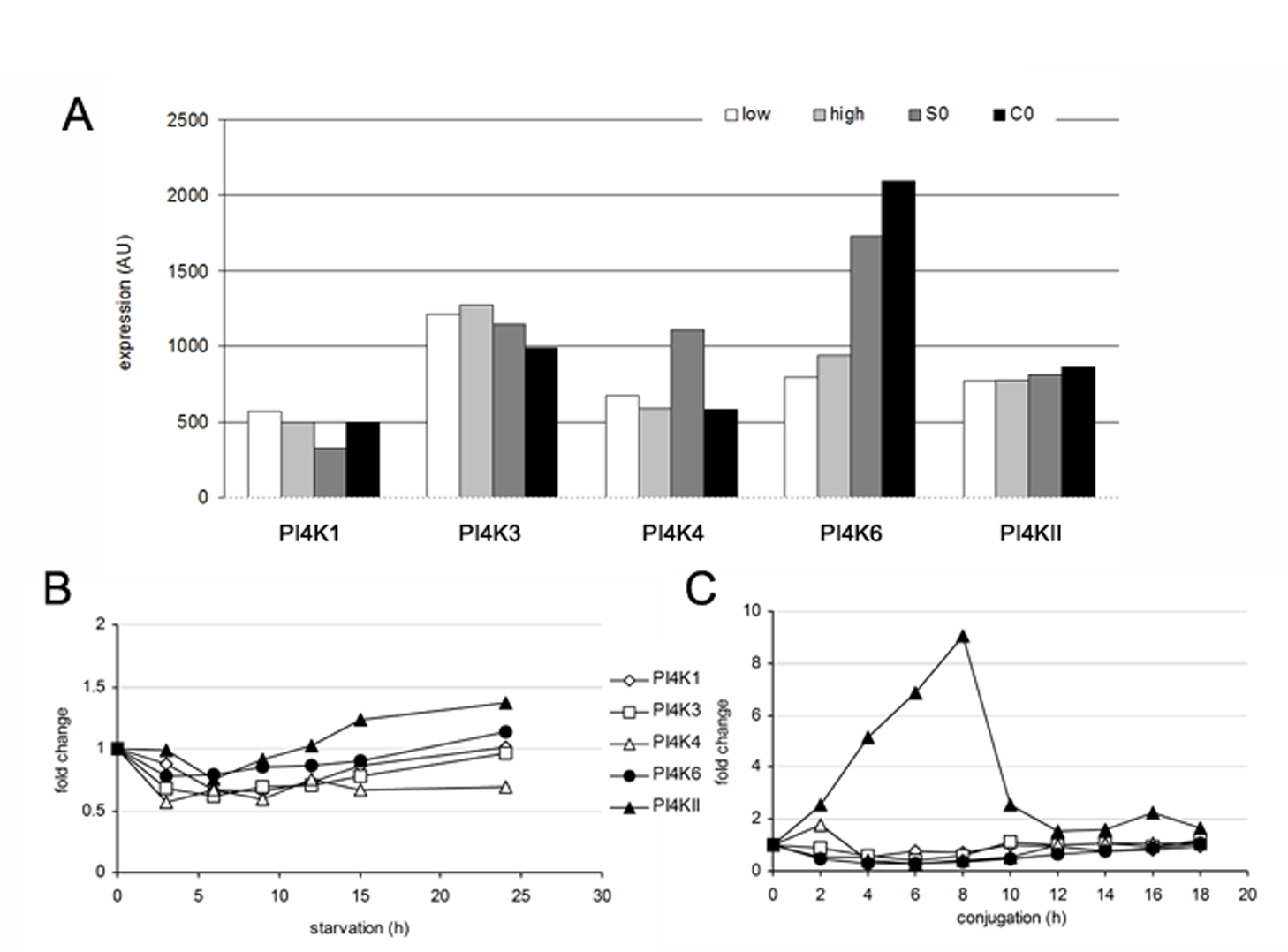

Supplement: Figure S1 — Expression patterns of Tetrahymena PI4K genes. A, Expression data for the indicated PI4K genes were extracted from the TGED site (http://tged.ihb.ac.cn/) and replotted in order to compare the expression at four different conditions: low and high cell density during growth, start of starvation (S0) and start of conjugation (C0). AU, arbitrary units. Expression data for PI4K2 and PI4K5 were unavailable, but both genes are expressed with at least one EST clone detected during starvation (reference 36 in the manuscript). B and C, Expression data during starvation and conjugation were normalized relative to controls (time 0) and are plotted as fold changes. Note the striking and gradual upregulation of TtPI4KII during conjugation reaching a peak (9-fold) at 8 h and the sharp decrease and stabilization after 10 h. (TIF) [file pone.0078848.s001.tif]

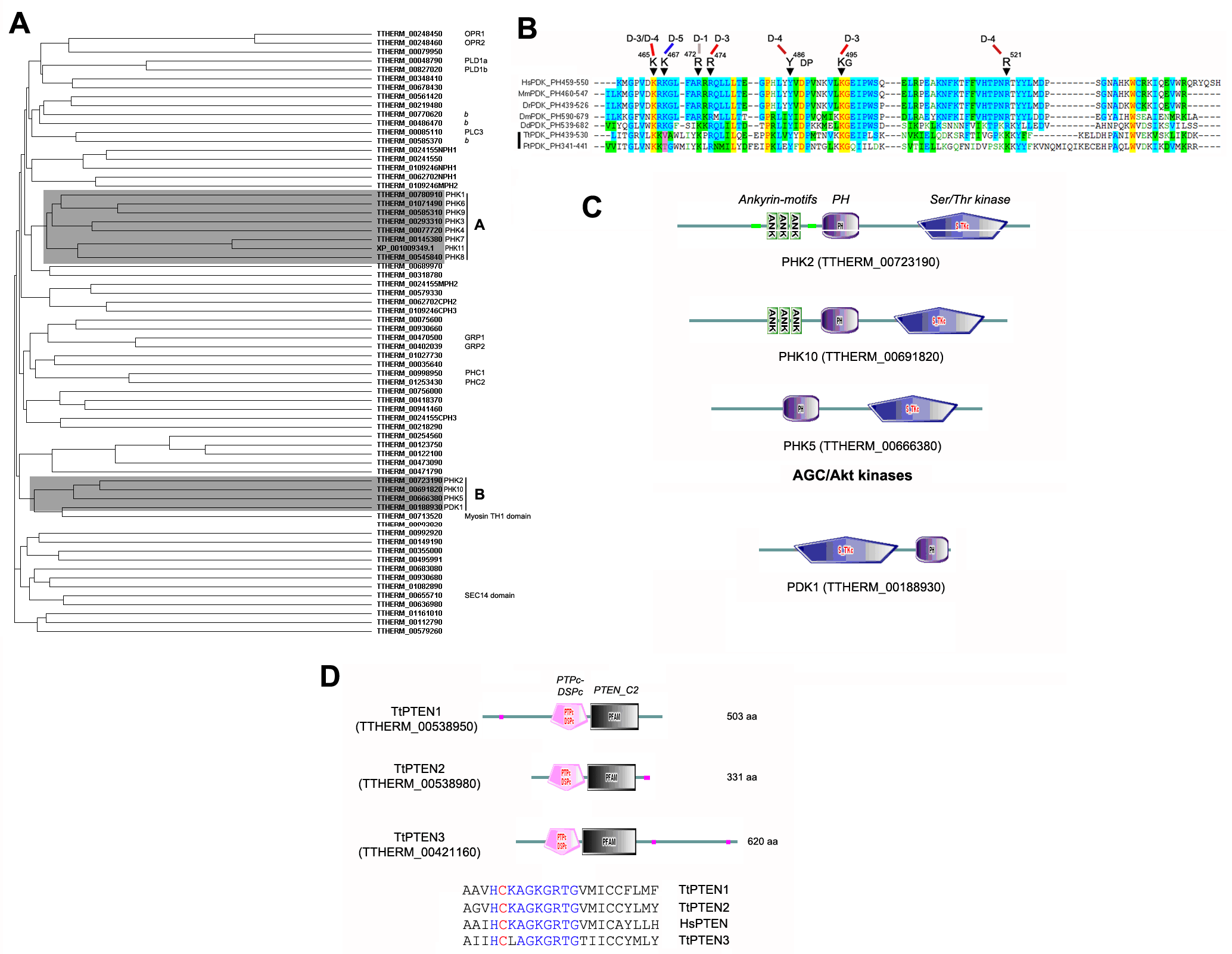

Supplement: Figure S2 — Components of a PI3K class I-associated PDK1-PKB/Akt-PTEN pathway are present in Tetrahymena . A, ClustalW-generated cladogram of a cohort of PH domains in T. thermophila gene products retrieved from the SMART database. The two grey boxes highlight two groups of PH-containing protein kinases (PHKs; these were numbered arbitrarily and their names are shown next to the respective locus tag). Group B consists of PHK2, PHK10, PHK5 and PDK1/PHK12 PH domains. Additional PH domains from other gene products, some of which are likely to be involved in various aspects of lipid/PI metabolism, are also indicated: OPR1,2 are oxysterol-binding protein (OSBP)-related proteins; PLD1a,b are phospholipases D; PLC3 is the inactive Tetrahymena PI-specific phospholipase C PRIP-like protein (reference 27 in the manuscript) and b indicates hits from BLAST analysis with the PLC3 PH domain that were included in the cohort; GRP1 is identical to the TtTST1 gene (a TBC-Sec7 family Arf-GEF) related to the previously described PH domain-containing TtGEF1 gene product (a GBF/BIG family Arf-GEF) (Bell et al., 2009, Cell Motil Cytoskeleton 66∶483–499; Awan et al., 2009, PLoS One 4(3):e4873); PHC1,2 are PH-containing adenylyl/guanylyl cyclases. PH-domain containing proteins with putative transmembrane regions, coiled-coil regions or low-scoring domains detected by SMART or PFAM databases are not indicated. B, Sequence alignments of PH domains from PDK1 orthologs reveals conservation of critical PtdIns(3,4,5)P3 D3/D4-phosphate interacting residues. Tetrahymena (and Paramecium) PDK1-PH domains retain key interactions with the D3-phosphate (residues K465 R474, K495; numbering refers to hPDK1 residues) and D4-phosphate (residues K465, Y486, R521) but not the D5-phosphate of PtdIns(3,4,5)P3 (residue K467 is replaced by V in TtPDK1) (reference 43 in the manuscript). The gene locus tag of PtPDK1 is GSPATT00036680001. PDK1 orthologs from Homo sapiens (Hs), Mus musculus (Mm), Danio rerio (Dr), Drosophila mel [file pone.0078848.s002.tif]

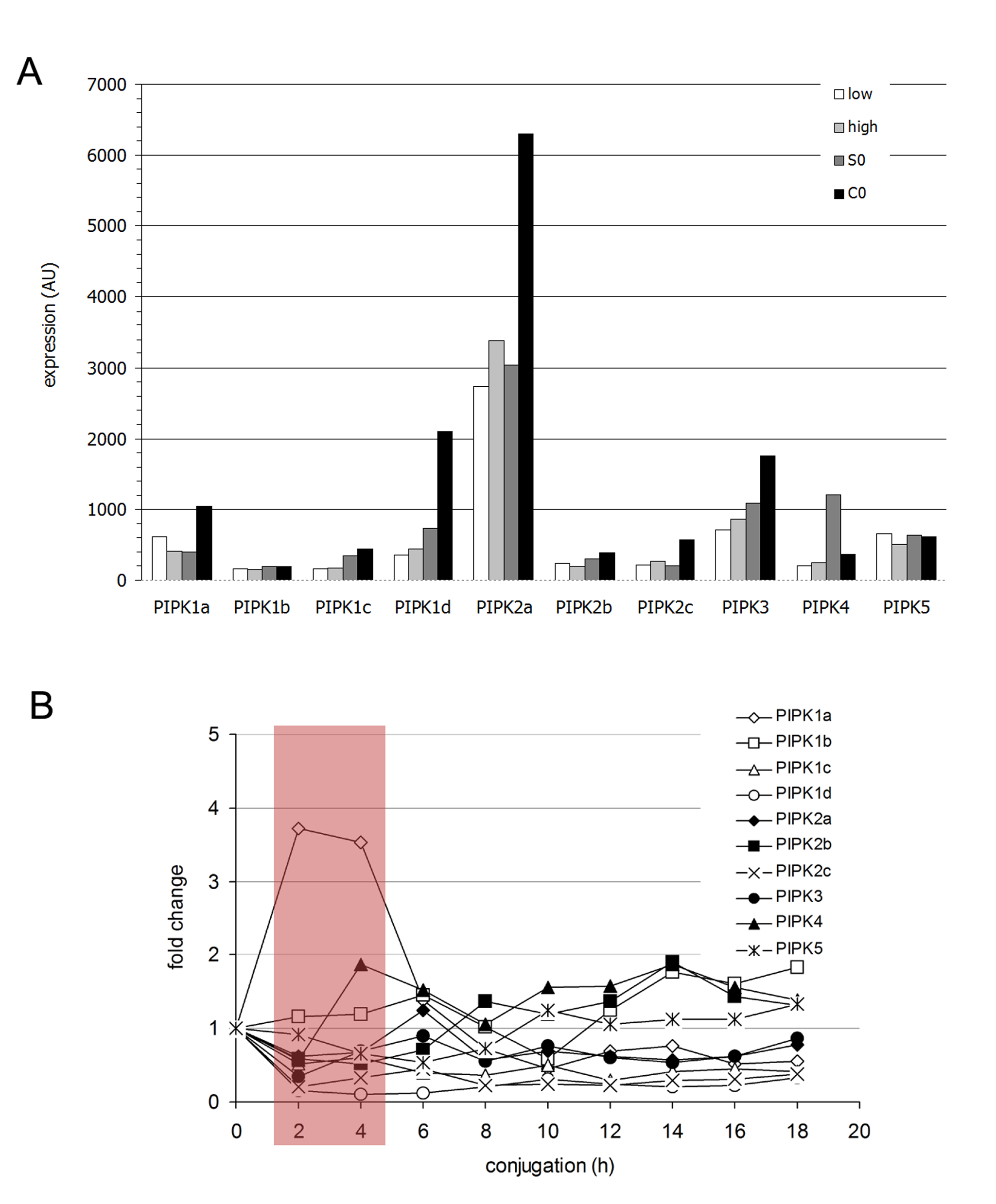

Supplement: Figure S3 — Expression patterns of Tetrahymena PIPK genes. A, Expression data for the indicated PIPK genes were extracted from the TGED site (http://tged.ihb.ac.cn/) and replotted in order to compare the expression at four different conditions: low and high cell density during growth, start of starvation (S0) and start of conjugation (C0). AU, arbitrary units. Note that TtPIPK1b,c, TtPIPK2b,c and TtPIPK4 are expressed at low levels during vegetative growth. B, Expression data during conjugation were normalized relative to controls (time 0) and are plotted as fold changes. The unique and transient upregulation of TtPIPK1a during 2–4 h of conjugation (3-4-fold) is highlighted by a box. (TIF) [file pone.0078848.s003.tif]

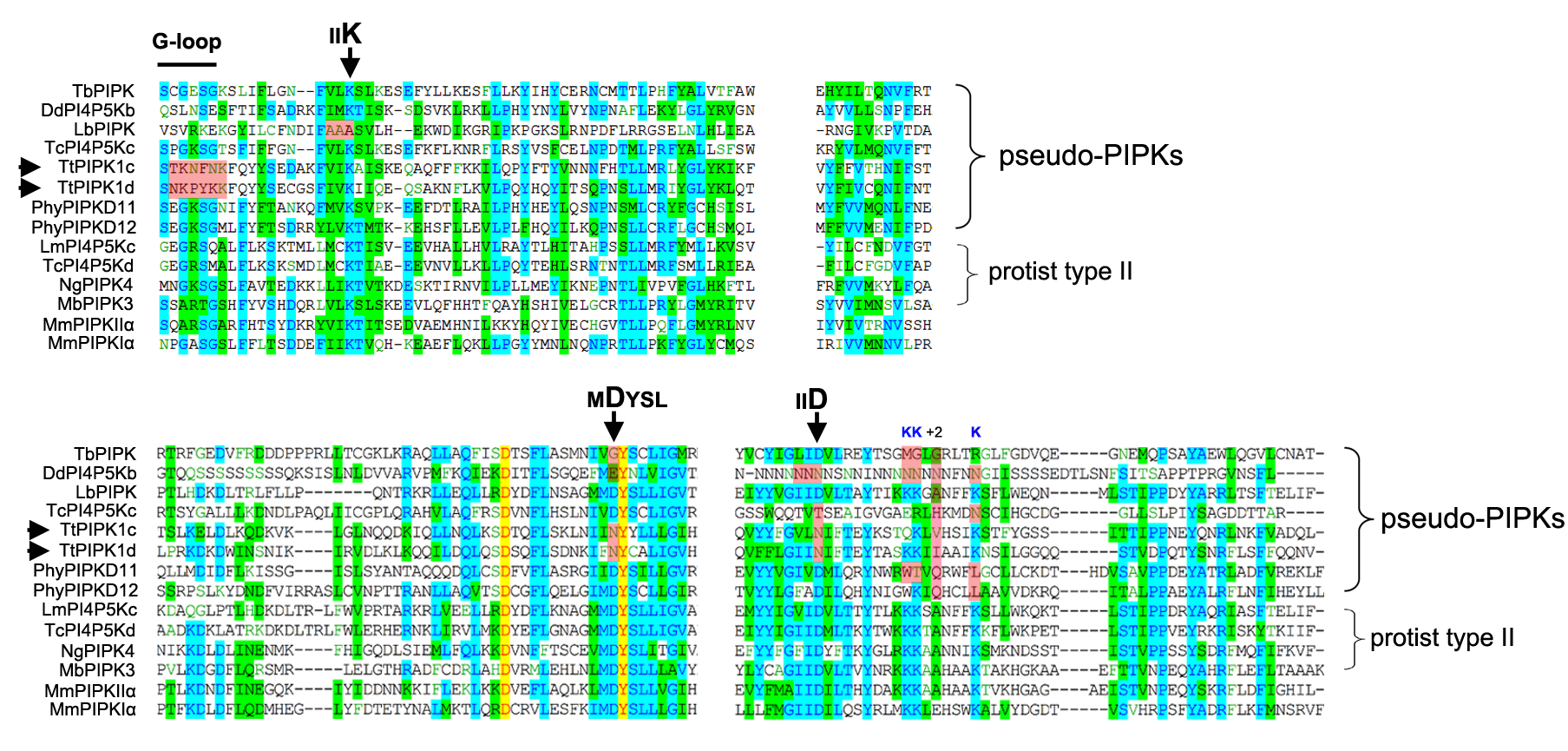

Supplement: Figure S4 — Occurrence of pseudo-PIPKs in protists. Substitution of key residues in the G-loop, IIK, MDYSL, IID motifs and/or the activation loop of the indicated pseudo-PIPKs is highlighted by red boxes. TtPIPK1c and TtPIPK1d are indicated by arrowheads. Four apparently active protist PIPKs that bear a type II activation loop (A residue in the +2 position) and representative mammalian type I and II PIPKs are also included in the alignment for comparison. Of the four protist type II PIPKs only the Monosiga brevicolis MbPIPK3 is phylogenetically related to metazoa PIPKII (see Figure 5). The accession numbers of PIPKs are listed in Table S3. (TIF) [file pone.0078848.s004.tif]
