# Supplementary material for: Genome-Wide Analysis of the Phosphoinositide Kinome from Two Ciliates Reveals Novel Evolutionary Links for Phosphoinositide Kinases in Eukaryotic Cells
Source: PLoS One. 2013 Nov 11;8(11):e78848. doi: 10.1371/journal.pone.0078848 (PMC3823935; doi:10.1371/journal.pone.0078848)
Supplement: Table S1 — Tetrahymena PIKs. (DOC) [file pone.0078848.s005.doc]

| **Locus tag**  Table S1. *Tetrahymena* PIKs | **Gene name** | **Genomic scaffold** | **Exons/ Introns** | **Domains (aa)** | **E value** | **Comments *^5^** |
| --- | --- | --- | --- | --- | --- | --- |
| **PI4Ks** | | | | | |
| **PI4K type III** | | | | | |
| TTHERM_00554370 | *TtPI4K1* | Scf_8254786 | 14/13 | PI3Ka (1553-1690)  PH (1720-1815) *^1^ PI3Kc (2022-2281) | 3.00e-07  - 5.25e-73 | Intact PIK (see comments) |
| **TTHERM_00219449** | *TtPI4K2* | Scf_8254564 | 14/13 | RING (265-305) PI3Ka (1133-1334)*^2^  PI3Kc (1494-1745) | 1.21e-03  - 5.78e-84 | 13/12, aa 867-1747, no RING domain, intact PIK |
| TTHERM_00157930 | *TtPI4K3* | Scf_8254776 | 10/9 | PI3Ka (274-452)*^2^  [PI3Kc](http://smart.embl-heidelberg.de/smart/do_annotation.pl?DOMAIN=PI3Kc&TYPE=SMART&START=588&END=839&LENGTH=251&E_VALUE=8.37726524223012e-81&BLAST=PFIIKGGDDLRQELLIMQLIQKFHEIFKQGGVPLYLRPYEIIVASESSGFIEFLPNTLSIDALKKKYPKFTNLYEFYRRTFNDYFEEAQKNFIESLAGYSLICYLLQIKDRHNGNILIDNQGHIIHIDFGFTLSIAPGGIKFETAHFKLTNEYVALMGGRNSDQFGYYKSLLVRAFIEIKKHVNTICNLVQIMCDQSNLPCFDEFDMIQFRGRFKENFTDKEFIDYVDELIKGSCDKWSTNQYDKFQLMQNG) (588-839) | -  8.38e-81 | 8/7, intact PIK |
| TTHERM_00219450 | *TtPI4K4* | scf_8254564 | 11/10 | PI3Ka (228-369)*^2^  PI3Kc (492-743) | -  9.21e-69 | 13/12, absence of aa 715-735 corresponding to the C-terminus of PIK domain (not included in the alignments) |
| TTHERM_00136219 | *TtPI4K5* | scf_8254582 | 10/9 | PI3Ka (252-452)*^2^  PI3Kc (600-862) | -  3.14e-62 | Verified gene model |
| TTHERM_00389990 | *TtPI4K6* | scf_8254587 | 12/11 | PI3Ka (339-492)*^2^  [PI3Kc](http://smart.embl-heidelberg.de/smart/do_annotation.pl?DOMAIN=PI3Kc&TYPE=SMART&START=619&END=891&LENGTH=272&E_VALUE=2.87840636321239e-70&BLAST=HFIVKTGDNLKQEQFAQQLIYQFDQIFKDSELNLILRPYEIISLGPNCGIIEVVKNSVTFDSLIQKLNTQQKGMNLQEFFKSYYGSGLYKAQKNFCSSLAAYSLVCYFLQIKDRHNGNILLHKDGYICHIDFGFLLSNAPGNNLIQKNNQRQGVGFELNVPFKLLTDYINVLGGVNSDLFKQFRQLFHKGFMAARKNQDKILILVKMFYSSHGASLPCFEKGEEAIKALEERFNPKNIKLENEYFVHTNQLIDQSLDNWKSRWYDKWQYFCQG) (619-891) | -  2.88e-70 | Verified gene model |
| **PI4K type II** | | | | | |  |
| TTHERM_00300180 | *TtPI4KII* | scf_8254379 | 4/3 | UBQ (19-96)  PI4IIK (111-484) | 6.05e-04  7.40e-23 | 3/2, absence of aa 326-374 in the kinase domain |
| **PI3Ks** | | | | | |  |
| **PI3Ks class I** | | | | | |  |
| TTHERM_00951960 | *TtPI3K1* | scf_8254466 | 12/11 | [PI3K_rbd](http://smart.embl-heidelberg.de/smart/do_annotation.pl?DOMAIN=PI3K_rbd&TYPE=SMART&START=147&END=244&LENGTH=97&E_VALUE=0.0435369248729924&BLAST=QEEKMIKDDKPIKYEKRIEIEAGLVTGADEVIKNILREIFKQAKVKLEPNNPDHQFILQIKGFREYLTGNYPMLSYDRVRINLRGHEHLDVRLTEIPI) (147-244)  [PI3K_C2](http://smart.embl-heidelberg.de/smart/do_annotation.pl?DOMAIN=PI3K_C2&TYPE=SMART&START=497&END=618&LENGTH=121&E_VALUE=0.0163881650145346&BLAST=LKLCKIRKEKLLKDAQMDTSITNHGIDGVAFMGGLEKKFGLNCTPYLVSLKVMLFHGSNCLRTVETKKQPFCKSIRFNEWVTFDNLKISQIPLEARICMNIILHSDDQNEHQIIGSTSFSLF) (497-618) [PI3Ka](http://smart.embl-heidelberg.de/smart/do_annotation.pl?DOMAIN=PI3Ka&TYPE=SMART&START=713&END=902&LENGTH=189&E_VALUE=4.19908349550448e-31&BLAST=NWNSTPKTDDLARLQQLLSIDPLRRLSEQSEEDKHVLMICRNHYKTLTHTLQIFLFAIDWLDPEQVKEAILMLKQWTLLSPEDALPLLDANMANESVRLYAVERVSTFSDDEIALYMLELTQSILYESKHFSPLVDMLLERSLQNPFVVGHELFWQLKSQLHIKAFYERYFLIIEQMLMLCGSFRNELVV) (713-902) [PI3Kc](http://smart.embl-heidelberg.de/smart/do_annotation.pl?DOMAIN=PI3Kc&TYPE=SMART&START=987&END=1248&LENGTH=261&E_VALUE=3.18396747466368e-104&BLAST=NILFKCGDDIRQDQLTLQLLKIMDKIWLDAGHDFRMKPYKVITTDDQVGMIEIVNNSETTAKIHSKYGGVLGAFRNNTIWQYLKDKNMDPHSFEIATDNFLRSCAGYCVATYILGIGDRHADNIMLSQTGHLFHIDFGHFLGNFKTKFGIKRERAPFVLTKEMAFVMGGKDGNLFRKFEEYCTQAYNLVRKSGNFIINIFLLMLEAGMPELQSPQDIEYLRNQLSINLTEQEATNKFKKEITDSLNSLFRRIDNFFHGLRRR) (987-1248) | 4.35e-02 1.64e-02 4.20e-31 3.18e-104 | Verified gene model |
| TTHERM_00655270 | *TtPI3K2* | scf_8254435 | 13/12 | [PI3K_rbd](http://smart.embl-heidelberg.de/smart/do_annotation.pl?DOMAIN=PI3K_rbd&TYPE=SMART&START=251&END=359&LENGTH=108&E_VALUE=0.111282731715727&BLAST=NKEKTTNNQPLEDQYSVKIKAELVTAFLYKLYFNKNKNRADEVLKKTLREVYYKYKIKLQPQDNKTFILKIEGYREYLSGHYPMLSYDRVRTNLRIKKSLKVILDQKQN) (251-359) [PI3K_C2](http://smart.embl-heidelberg.de/smart/do_annotation.pl?DOMAIN=PI3K_C2&TYPE=SMART&START=822&END=925&LENGTH=103&E_VALUE=0.00548726675774236&BLAST=FATDHGDKGCSYFQQLCQKYDINFAPYLIQVQVKLMYGNRALAVKKETQKIPFSRSPRWDEWIKFPLIKISQLPLESRICFDIIVYSVTGLDSQIIGSTNFYIF) (822-925) [PI3Ka](http://smart.embl-heidelberg.de/smart/do_annotation.pl?DOMAIN=PI3Ka&TYPE=SMART&START=1029&END=1211&LENGTH=182&E_VALUE=1.69711121476081e-17&BLAST=PQNQYLAELKELLKRDPINRNFDMRQKLILLICRNHFKSIHQALDIFLCAVDWSNPEHIRETYNLLKQWKSPPCEECITFLDAHHADEVIRLYGVERISKMADDEIKLYMIELVQALMFENNHYSPIQELLLERSLLNPFVVGHELFWSLKSQLHLKQSYERYALVLEQLLMLSGEYRSELLK) (1029-1211) [PI3Kc](http://smart.embl-heidelberg.de/smart/do_annotation.pl?DOMAIN=PI3Kc&TYPE=SMART&START=1302&END=1559&LENGTH=257&E_VALUE=1.54715369923938e-93&BLAST=SIMFKDGDDLRQDILTLQLIRIMDKIWLDNNLDLRMKPYNVVATLDQVGMLEIVQNSQTTAAIHKQNNQRFGCLMKESIQKYLKKHNKNYEQARANFRTSCAGYCVATYILGIGDRHSDNIMITKEGHFFHIDFGHFLGNFKKKFGINRERSPFVFTRVMEYVMLEKQEDYEAFEKYCCQAYNLIRKHGNFFINIFRMMLSAGMPELQRESDIEYLVETLQLNLSDQEAEQHFKKQIRIAREDKFKLFDNFIHEIVKK) (1302-1559) | 1.11e-01 5.49e-03 1.70e-17 1.55e-93 | 14/13, 568-642 aa alternative sequence, intact PIK domain |
| TTHERM_00323020 | *TtPI3K3* | scf_8254691 | 13/12 | [PI3K_rbd](http://smart.embl-heidelberg.de/smart/do_annotation.pl?DOMAIN=PI3K_rbd&TYPE=SMART&START=111&END=217&LENGTH=106&E_VALUE=0.00595932910958568&BLAST=LNVNLDEYINQLREKKPAEVDWDCKSLTIEAGLVTGADEVIKNILRKIYKEYKIALKPKDQNHQFILQVKGFREYLAGNHPMLNYDRVRTSLRGLDHLDVKLTEMPI) (111-217) [PI3K_C2](http://smart.embl-heidelberg.de/smart/do_annotation.pl?DOMAIN=PI3K_C2&TYPE=SMART&START=395&END=523&LENGTH=128&E_VALUE=0.0464065280405563&BLAST=KQNKDIQLQQEQQKQRKDQVTPKLNTRIVNHGIKGVEFMKQIQGYYGMNLVPFSISIEAIIMNGSTCIKSVETKRVPYGNTCKFNQWISFNNIKVSQLPLESRLCFNLKAYAQNSNDYQIIGSSTLPIF) (395-523) [PI3Ka](http://smart.embl-heidelberg.de/smart/do_annotation.pl?DOMAIN=PI3Ka&TYPE=SMART&START=622&END=812&LENGTH=190&E_VALUE=1.19564314839166e-35&BLAST=HRNFNQTPKTEDLARLQFLLNADPLKRPEFTPEDKHILMISRNHYKTLTQALQVFLLAVDWLDPEQVKEAILMLKKWTPLQPEDALPLLDAHISNEIVRLYAVERISTFSDDEIALYMLELTQLLLYERNHFSPLAEMLLERSLLNPYVVGHEFFWQLKSQLNVKAFHERFSLLIEQFIMVCGSFRKEIAN) (622-812) [PI3Kc](http://smart.embl-heidelberg.de/smart/do_annotation.pl?DOMAIN=PI3Kc&TYPE=SMART&START=939&END=1200&LENGTH=261&E_VALUE=2.54250459099113e-112&BLAST=KILFKRGDDIRQDQLTLQLLKIMDKIWLDAGQDFRMKPYKVITTGDQVGMIEVVVNAETTARIHKQEGGGLGALKEDTLKNYLQKHNPDKENLKSATENFIRSCAGYCVATYILGIGDRHPDNIMLSQTGHLFHIDFGHFLGNFKKKLGIDRERAPFVFTEEMAFVMGGKESADFKNFTDYCTQAYNLIRKQGNFIINIFLLMLDAGIPELQSAKNIEYLKNKLALQLSEQEATNKFKKEIHFSLNSRFRKLDNLIHNLVKQ) (939-1200) | 5.96e-03 4.64e-02 1.20e-35 2.54e-112 | 14/13, intact PIK |
| **PI3Ks class III** | | | | | |  |
| TTHERM_00649380 | *TtPI3KIII* | scf_8254644 | 6/5 | [PI3K_C2](http://smart.embl-heidelberg.de/smart/do_annotation.pl?DOMAIN=PI3K_C2&TYPE=SMART&START=21&END=122&LENGTH=101&E_VALUE=0.790114531597709&BLAST=FKICCINDQKKVLRYFFNDEDGSSKLENLEAMVTAQLVVNEKKIQPAQQTLFIDRMLFEQNIIFNYKYKDLSYNSIIAITLWCSQKNKDEKKPLGCTTISLF) (21-122) [PI3Ka](http://smart.embl-heidelberg.de/smart/do_annotation.pl?DOMAIN=PI3Ka&TYPE=SMART&START=306&END=515&LENGTH=209&E_VALUE=1.74308516775748e-37&BLAST=PQQLKPSKNELQIIENSIKKPILGELKQDEKLLFYRFRYSLVQNPEALVKFLHSINWDNQKELNEAKKLLKIWAKCSYGDALHLLSSSFCANEFYNKKYKKPQDTAIYIRKYACEQLEKEQTSTICSILLQLTQALRYEPFDYQKSHLAQFLVKKACENTEIATLFYWYLLVECDKEMDLKEKAKLSSTNQKVQEWFNQVFERMNEELEE) (306-515) [PI3Kc](http://smart.embl-heidelberg.de/smart/do_annotation.pl?DOMAIN=PI3Kc&TYPE=SMART&START=617&END=888&LENGTH=271&E_VALUE=4.31791711125227e-83&BLAST=EMIYKNGDDLRQDQLVMQIFNLMDGLLKGVGQDFRLMPYKVLACSKNDGFMEFVPNSTTIQDLLKGGKQFSQYLQNLAANPENPIYEEYKRLRMQNDEQGKLSNIEYVSQKIMENYMLSCAGYSVMTYFLGVGDRHLENLMVDITGKFFHIDFGFILGQDPKPYPPPLKLCRQMIEGMGGKDSKVYAEFRKKCVHAYIYLRKYAKLIVNLFHLMIDSGIKDMSIEALEKLAEKFYLDFNDTQAELHFLNILDESETALFARVTDMIHKWATY) (617-888) | 7.90e-01 1.74e-37 4.32e-83 | Verified gene model |
| **PIPKs** | | | | | |  |
| TTHERM_01125130 | *TtPIPK1a* | scf_8254380 | 10/9 | EFh (169-204)  EFh (220-255)  PIP5K (1140-1401) | 0.14 *^3^  0.0094 *^3^  3.80e-71 | smaller size of exon 3, intact EFh and PIK |
| TTHERM_01457080 | *TtPIPK1b* | scf_8253893 | 11/10 | [EFh](http://smart.embl-heidelberg.de/smart/do_annotation.pl?DOMAIN=EFh&TYPE=SMART&START=151&END=179&LENGTH=28&E_VALUE=16.8433807536425&BLAST=FVDRIFKLMDENGTEKAVFEDYLRYLSQL) (151-179) [EFh](http://smart.embl-heidelberg.de/smart/do_annotation.pl?DOMAIN=EFh&TYPE=SMART&START=151&END=179&LENGTH=28&E_VALUE=16.8433807536425&BLAST=FVDRIFKLMDENGTEKAVFEDYLRYLSQL) (232-260) PIP5K (675-864) | 1.68e+01  2.14e-01  2.00e-68 | 13/12, additional exon and larger exon in PIK domain results in insertions at 739, 761 and 816 aa (all major catalytic features are present) |
| TTHERM_00600820 | *TtPIPK1c* | scf_8254563 | 7/6 | PIP5K (518-814) | 2.10e-53 | 5/4, intact PIK |
| TTHERM_01075790 | *TtPIPK1d* | scf_8254412 | 1/- | [EFh](http://smart.embl-heidelberg.de/smart/do_annotation.pl?DOMAIN=EFh&TYPE=SMART&START=86&END=114&LENGTH=28&E_VALUE=10.3659540402059&BLAST=LADRIFDLIDSDKNEWISFMDFLKYLDVI) (86-114) [EFh](http://smart.embl-heidelberg.de/smart/do_annotation.pl?DOMAIN=EFh&TYPE=SMART&START=86&END=114&LENGTH=28&E_VALUE=10.3659540402059&BLAST=LADRIFDLIDSDKNEWISFMDFLKYLDVI) (122-150) [EFh](http://smart.embl-heidelberg.de/smart/do_annotation.pl?DOMAIN=EFh&TYPE=SMART&START=86&END=114&LENGTH=28&E_VALUE=10.3659540402059&BLAST=LADRIFDLIDSDKNEWISFMDFLKYLDVI) (168-196) PIP5K (1310-1607) | 1.04e+01  7.36e+00  1.83e+01  2.60e-45 | ambiguous EFh, intact PIK |
| TTHERM_00149640 | *TtPIPK2a* | scf_8254545 | 2/1 | TMs (55-76,89-111,131-153,166-184,204-226,247-269,279-301) *^4^  PIP5K (485-744) | 1.10e-55 | Verified gene model |
| TTHERM_00310510 | *TtPIPK2b* | scf_8254551 | 11/10 | [RING](http://smart.embl-heidelberg.de/smart/do_annotation.pl?DOMAIN=RING&TYPE=SMART&START=352&END=392&LENGTH=40&E_VALUE=6.23651961876392e-06&BLAST=CMICLTDFEESNLCRMTVCYHLFHKNCLESWLELQDSCPFC) (352-392)  TMs (870-892,899-921,936-958,979-998,1018-1040,1061-1080)  PIP5K (1250-1511) | 6.24e-06  6.50e-59 | 3/2, absence of RING, preservation of 7 TMs and intact PIK |
| TTHERM_00047210 | *TtPIPK2c* | scf_8254659 | 5/4 | TMs (57-79,91-113,133-155,176-198,208-228,249-271)  PIP5K (480-756) | 5.20e-54 | 4/3, preservation of 7TMs, larger last exon results in reconstitution of the missing DLKGS motif in the PIK domain |
| TTHERM_01005090 | *TtPIPK3* | scf_8254757 | 9/8 | [FYVE](http://smart.embl-heidelberg.de/smart/do_annotation.pl?DOMAIN=FYVE&TYPE=SMART&START=38&END=111&LENGTH=73&E_VALUE=4.54908178187816e-14&BLAST=VKPQAWKKDEDSKLCFICQKEFNFLYRRRHHCRKCANLFCSSCSNYFLSLLFNPLDEVDKETRLCKNCFDQFKQ) (38-111)  Cpn60_TCP1 (272-500)  PIP5K (1503-1721) | 4.55e-14  6.70e-08  2.50e-48 | 10/9, intact FYVE, TCP1, PIK domains |
| TTHERM_00467410 | *TtPIPK4* | scf_8254371 | 3/2 | PIP5K (113-401) | 4.20e-40 | 2/1, aa 1-396, intact PIK domain |
| TTHERM_00530790 | *TtPIPK5* | scf_8254459 | 11/10 | Plug_translocon (53-87)  SecY (88-472)  PIP5K (1605-1877) | 4.50e-21  5.10e-81  1.70e-64 | 1/-, aa 792-1879, absence of translocon and SecY domains, intact PIK |
| TTHERM_00471898 | *TtPIPK6* | scf_8254565 | 8/7 | PIP5K (1570-1841) | 8.60e-68 | 6/5, intact PIK |

*^1^ identified by sequence alignments with STT4 orthologs from *H. sapiens*, *A. thaliana* and *S. cerevisiae*.

*^2^ identified as a region of similarity to the PI3Ka domain of TtPI4K1 and TtPI3Ks in sequence alignments

*^3^ EFh regions in TtPIPK1a were detected by Prosite

*^4^ TMs, transmembrane regions

*^5^ Corrections suggested after comparison with RNA deep sequencing results of Xiong et al., 2012 at the TetraFGD site. Given are the alternative exon/intron numbers, verification of identified domains in the original gene models and whether the PIK domain is intact. However, the amino acid numbers refer to the original gene models available at the *Tetrahymena* gene database. For some PIK genes, TetraFGD predicts multiple transcription units within the same gene; however, examination of intron-exon boundaries and base-coverage plots revealed a unique ORF corresponding to the original PIK. Examples of such cases are PI3K2, PIPK1b, PIPK2b, PIPK1c, PIPK2c, PI4K3 but not PI4K1 which appears to code for a shorter, 1168 aa, protein. Given the high homology of PI4K1 with the *Paramecium* STT4 homolog throughout the amino acid sequence (ranging from 52 % in the N-terminal half of PI4K1 to 83% in the catalytic domain), we verified the gene model of *Paramecium* STT4 using RNA sequencing data: indeed, it encodes a large protein with the expected size with very minor changes compared to the original gene model (Arnaiz, O., personal communication).
