# Supplementary material for: Genome-Wide Analysis of the Phosphoinositide Kinome from Two Ciliates Reveals Novel Evolutionary Links for Phosphoinositide Kinases in Eukaryotic Cells
Source: PLoS One. 2013 Nov 11;8(11):e78848. doi: 10.1371/journal.pone.0078848 (PMC3823935; doi:10.1371/journal.pone.0078848)
Supplement: Table S2 — Paramecium PIKs. (DOC) [file pone.0078848.s006.doc]

| **Locus tag**  Table S2. *Paramecium* PIKs | **Gene name (PIK class or type)** | **Genomic scaffold** | **Exons/ Introns** | **Domains (aa)** | **E value** |
| --- | --- | --- | --- | --- | --- |
| **PI4Ks** | | | | | |
| **PI4K type III** | | | | | |
| GSPATT00029485001 | PtStt4/ IIIα | scaffold_111 | 5/4 | PI3Kc (2060-2324) | 6.32e-67 |
| GSPATT00006568001 | PtPI4K1 / ΙΙΙβ | scaffold_16 | 6/5 | PI3Kc (569-820) | 1.30e-68 |
| GSPATT00002101001 | PtPI4K2 / ΙΙΙβ | scaffold_5 | 6/5 | PI3Kc (569-820) | 9.87e-69 |
| GSPATT00007965001 | PtPI4K3 / ΙΙΙβ | scaffold_20 | 5/4 | PI3Kc (473-726) | 1.22e-82 |
| GSPATT00027213001 | PtPI4K4 / ΙΙΙβ | scaffold_98 | 9/8 | PI3Kc (442-694) | 1.00e-78 |
| GSPATT00004100001 | PtPI4K5 / ΙΙΙβ | scaffold_9 | 8/7 | PI3Kc (440-700) | 1.03e-71 |
| GSPATT00023634001 | PtPI4K6 / ΙΙΙβ | scaffold_79 | 6/5 | PI3Kc (501-749) | 2.44e-59 |
| GSPATT00010804001 | PtPI4K7 / ΙΙΙβ | scaffold_29 | 6/5 | PI3Kc (490-744) | 4.54e-68 |
| GSPATT00025087001 | PtPI4K8 / ΙΙΙβ | scaffold_86 | 6/5 | PI3Kc (324-576) | 3.96e-59 |
| GSPATT00032462001 | PtPI4K9 / ΙΙΙβ | scaffold_130 | 7/6 | [PI3Kc](http://smart.embl-heidelberg.de/smart/do_annotation.pl?DOMAIN=PI3Kc&TYPE=SMART&START=353&END=605&LENGTH=252&E_VALUE=5.10705863035289e-53&BLAST=QIIIKGGDDLRNELLIMQMIKKIKEIFEKHKLDIFLRPYDIILTSPNSGILEFIPNTVSIDKIKRDYQDYTLKQFYENFFDCFSEAQTNFLQSLAGYSLICYLFQLKDRHNGNILIDNQGHIIHIDFGFVLTLTPGNLGFETAPFKLIEEYEELLDGKGSYFHNYFQTLIFKGLMALQQEIDEILNFVKIMNFSPKHNLLVSLEKFKIEDFRKRFMEKCSLEQLYENVQNIVKESSNSWTTTLYDYFQFKTNT) (353-605) | 5.11e-53 |
| GSPATT00036199001 | PtPI4K10 / ΙΙΙβ | scaffold_158 | 7/6 | PI3Kc (343-595) | 2.68e-47 |
| GSPATT00020399001 | PtPI4K11 / ΙΙΙβ | scaffold_64 | 6/5 | PI3Kc (419-662) | 3.53e-47 |
| GSPATT00005667001 | PtPI4K12 / ΙΙΙ-like | scaffold_13 | 5/4 | PI3Kc (560-823) | 8.21e-66 |
| GSPATT00035938001 | PtPI4K13 / ΙΙΙ-like | scaffold_156 | 5/4 | PI3Kc (553-816) | 6.37e-63 |
| GSPATT00025827001 | PtPI4K14 / ΙΙΙ-like | scaffold_90 | 7/6 | PI3Kc (431-692) | 9.15e-64 |
| GSPATT00023118001 | PtPI4K15 / ΙΙΙ-like | scaffold_77 | 8/7 | PI3Kc (425-686) | 1.52e-60 |
| GSPATT00006341001 | PtPI4K16 / ΙΙΙ-like | scaffold_15 | 5/4 | PI3Kc (434-685) | 2.45e-73 |
| GSPATT00009663001 | PtPI4K17 / ΙΙΙ-like | scaffold_25 | 4/3 | PI3Kc (434-685) | 5.84e-71 |
| PTMB.108c | PtPI4K18 / ΙΙΙ-like | - | 3/2 | PI3Kc (484-755) | 2.93e-53 |
| GSPATT00011560001 | PtPI4K19 / ΙΙΙ-like | scaffold_31 | 6/5 | PI3Kc (450-705) | 6.93e-64 |
| GSPATT00011117001 | PtPI4K20 / ΙΙΙ-like | scaffold_30 | 9/8 | PI3Kc (454-710) | 8.95e-58 |
| GSPATT00000433001 | PtPI4K21 / ΙΙΙ-like | scaffold_1 | 6/5 | PI3Kc (484-729) | 1.09e-52 |
| **PI4K type II** | | | | | |
| GSPATT00010125001 | PtPI4KIIa | scaffold_26 | 7/6 | UBQ (23-95)  PI3_PI4_kinase (144-408) | 5.87e-02  3.50e-26 |
| GSPATT00004156001 | PtPI4KIIb | scaffold_9 | 8/7 | UBQ (31-103)  PI3_PI4_kinase (152-416) | 1.69e-02  7.20e-29 |
| GSPATT00030531001 | PtPI4KIIc | scaffold_117 | 7/6 | UBQ (31-111)  PI3_PI4_kinase (160-424) | 0.00e+00  2.90e-28 |
| **PI3Ks** | | | | | |
| GSPATT00039649001 | PtPI3K1 / Ia | scaffold_556 | 2/1 | PI3K_rbd (120-227)  PI3K_C2 (457-581)  PI3Ka (646-836)  PI3Kc (959-1225) | 2.97e-01  5.6e-09  2.18e-29  3.12e-89 |
| GSPATT00018694001 | PtPI3K2 / Ia | scaffold_57 | 3/2 | PI3K_rbd (87-219)  PI3K_C2 (454-563)  PI3Ka (829-1017)  PI3Kc (1162-1422) | 7.62e-03  2.73e-02  1.80e-30  7.11e-93 |
| GSPATT00012877001 | PtPI3K3 / Ia | scaffold_35 | 4/3 | PI3K_rbd (128-260)  PI3K_C2 (503-603)  PI3Ka (859-1047)  PI3Kc (1185-1445) | 2.85e-03  2.00e-02  3.36e-30  9.10e-91 |
| GSPATT00027505001 | PtPI3K4 / Ia | scaffold_99 | 3/2 | PI3K_rbd (77-218)  PI3K_C2 (457-566)  PI3Ka (832-1020)  PI3Kc (1166-1426) | 3.40e-02  4.15e-02  8.27e-30  6.90e-91 |
| GSPATT00019938001 | PtPI3K5 / Ia | scaffold_62 | 3/2 | PI3K_rbd (99-200)  PI3K_C2 (335-443)  PI3Ka (634-822)  PI3Kc (915-1175) | 1.67e-02  2.94e-03  1.02e-29  1.31e-91 |
| GSPATT00022039001 | PtPI3K6 / III | scaffold_72 | 5/4 | PI3K_C2 (22-120)  PI3Ka (296-490)  PI3Kc (583-868) | 1.60e-02  7.26e-26  5.36e-79 |
| **PIPKs** | | | | | |
| GSPATT00000655001 | PtPIPK1a / I-II | scaffold_2 | 3/2 | [EFh](http://smart.embl-heidelberg.de/smart/do_annotation.pl?DOMAIN=EFh&TYPE=SMART&START=66&END=94&LENGTH=28&E_VALUE=2.2821111001694&BLAST=LGDALFQAMDINLDGHIEFEEFVAYFDKI) (66-94)  [EFh](http://smart.embl-heidelberg.de/smart/do_annotation.pl?DOMAIN=EFh&TYPE=SMART&START=66&END=94&LENGTH=28&E_VALUE=2.2821111001694&BLAST=LGDALFQAMDINLDGHIEFEEFVAYFDKI) (102-130)  [EFh](http://smart.embl-heidelberg.de/smart/do_annotation.pl?DOMAIN=EFh&TYPE=SMART&START=66&END=94&LENGTH=28&E_VALUE=2.2821111001694&BLAST=LGDALFQAMDINLDGHIEFEEFVAYFDKI) (153-181)  PIP5K (429-674) | 2.28e+00  7.36e+00  7.08e-01  1.40e-70 |
| GSPATT00005572001 | PtPIPK1b / I-II | scaffold_13 | 4/3 | [EFh](http://smart.embl-heidelberg.de/smart/do_annotation.pl?DOMAIN=EFh&TYPE=SMART&START=50&END=78&LENGTH=28&E_VALUE=0.26399463827081&BLAST=LAERIFNQIDGNKDGVIMFEDFVKYLSML) (50-78)  [EFh](http://smart.embl-heidelberg.de/smart/do_annotation.pl?DOMAIN=EFh&TYPE=SMART&START=50&END=78&LENGTH=28&E_VALUE=0.26399463827081&BLAST=LAERIFNQIDGNKDGVIMFEDFVKYLSML) (131-159)  PIP5K (463-709) | 2.64e-01  7.07e-02  5.20e-58 |
| GSPATT00006945001 | PtPIPK1c / I-II | scaffold_17 | 5/4 | PIP5K (412-683) | 9.00e-69 |
| GSPATT00019649001 | PtPIPK1d / I-II | scaffold_61 | 4/3 | PIP5K (550-810) | 3.10e-68 |
| GSPATT00032331001 | PtPIPK1e / I-II | scaffold_129 | 4/3 | [EFh](http://smart.embl-heidelberg.de/smart/do_annotation.pl?DOMAIN=EFh&TYPE=SMART&START=64&END=92&LENGTH=28&E_VALUE=1.36493077251938&BLAST=LSDCLFQIIDEDQDGKIQFNEFLAYFDKI) (64-92)  [EFh](http://smart.embl-heidelberg.de/smart/do_annotation.pl?DOMAIN=EFh&TYPE=SMART&START=64&END=92&LENGTH=28&E_VALUE=1.36493077251938&BLAST=LSDCLFQIIDEDQDGKIQFNEFLAYFDKI) (151-179)  PIP5K (516-767) | 1.36e+00  6.88e-01  1.90e-67 |
| GSPATT00000714001 | PtPIPK1f / I-II | scaffold_2 | 4/3 | [EFh](http://smart.embl-heidelberg.de/smart/do_annotation.pl?DOMAIN=EFh&TYPE=SMART&START=64&END=92&LENGTH=28&E_VALUE=1.36493077251938&BLAST=LSDCLFQIIDEDQDGKIQFNEFLAYFDKI) (64-92)  [EFh](http://smart.embl-heidelberg.de/smart/do_annotation.pl?DOMAIN=EFh&TYPE=SMART&START=64&END=92&LENGTH=28&E_VALUE=1.36493077251938&BLAST=LSDCLFQIIDEDQDGKIQFNEFLAYFDKI) (151-179)  PIP5K (515-766) | 1.36e+00  1.40e+00  5.20e-68 |
| GSPATT00010565001 | PtPIPK1g / I-II | scaffold_28 | 4/3 | [EFh](http://smart.embl-heidelberg.de/smart/do_annotation.pl?DOMAIN=EFh&TYPE=SMART&START=67&END=95&LENGTH=28&E_VALUE=3.92612232507076&BLAST=LSDRIFHIMDDDKDGKIRFEDFALYFDKV) (67-95)  [EFh](http://smart.embl-heidelberg.de/smart/do_annotation.pl?DOMAIN=EFh&TYPE=SMART&START=67&END=95&LENGTH=28&E_VALUE=3.92612232507076&BLAST=LSDRIFHIMDDDKDGKIRFEDFALYFDKV) (103-131)  [EFh](http://smart.embl-heidelberg.de/smart/do_annotation.pl?DOMAIN=EFh&TYPE=SMART&START=67&END=95&LENGTH=28&E_VALUE=3.92612232507076&BLAST=LSDRIFHIMDDDKDGKIRFEDFALYFDKV) (154-182)  PIP5K (552-812) | 3.93e+00  6.26e+01  9.61e+01  1.50e-67 |
| GSPATT00006938001 | PtPIPK1h / I-II | scaffold_17 | 6/5 | PIP5K (424-668) | 2.80e-67 |
| GSPATT00014456001 | PtPIPK2a / IV | scaffold_41 | 4/3 | 6 TMs *^1^ PIP5K (445-687) | 1.60e-59 |
| GSPATT00010975001 | PtPIPK2b / IV | scaffold_29 | 4/3 | 7 TMs *^1^ PIP5K (434-687) | 5.50e-56 |
| GSPATT00004271001 | PtPIPK2c / IV | scaffold_10 | 2/1 | 7 TMs *^1^ PIP5K (389-635) | 7.90e-53 |
| GSPATT00019508001 | PtPIPK2d / IV | scaffold_61 | 4/3 | 7 TMs *^1^ PIP5K (468-714) | 9.90e-58 |
| GSPATT00035744001 | PtPIPK2e / IV | scaffold_154 | 1/- | 7 TMs *^1^  PIP5K (403-646) | 2.00e-52 |
| GSPATT00014408001 | PtPIPK2f / IV | scaffold_41 | 6/5 | 7 TMs *^1^  PIP5K (464-721) | 2.30e-14  7.80e-57 |
| GSPATT00014678001 | PtPIPK2g / IV | scaffold_42 | 5/4 | 5 TMs *^1^  PIP5K (466-722) | 1.50e-54 |
| GSPATT00010924001 | PtPIPK2h / IV | scaffold_29 | 8/7 | 7 TMs *^1^  PIP5K (472-729) | 1.80e-58 |
| GSPATT00023769001 | PtPIPK2i / IV | scaffold_80 | 3/2 | 7 TMs *^1^  PIP5K (442-705) | 3.70e-50 |
| GSPATT00036728001 | PtPIPK2j / IV | scaffold_162 | 3/2 | 7 TMs *^1^  PIP5K (442-705) | 3.20e-51 |
| GSPATT00006031001 | PtPIPK2k / IV | scaffold_14 | 5/4 | 7 TMs *^1^  PIP5K (453-716) | 2.00e-53 |
| GSPATT00020951001 | PtPIPK2l / IV | scaffold_67 | 4/3 | 6 TMs *^1^  PIP5K (449-710) | 4.30e-49 |
| GSPATT00036767001 | PtPIPK2m / IV | scaffold_163 | 5/4 | 7 TMs *^1^  PIP5K (781-1061) | 1.50e-51 |
| GSPATT00029147001 | PtPIPK2n / IV | scaffold_109 | 4/3 | 8 TMs *^1^  PIP5K (467-733) | 2.10e-46 |
| GSPATT00030931001 | PtPIPK2o / IV | scaffold_120 | 5/4 | 8 TMs *^1^  PIP5K (461-727) | 1.10e-49 |
| GSPATT00010139001 | PtPIPK3a /III | scaffold_26 | 5/4 | [FYVE](http://smart.embl-heidelberg.de/smart/do_annotation.pl?DOMAIN=FYVE&TYPE=SMART&START=7&END=82&LENGTH=75&E_VALUE=1.19830914865118e-22&BLAST=LQTTKWVQDRDAKACKKCQNPFKAIFRRKHHCRNCGQLFCDSCSNYFMDKTNFKNYQEIKKNKVRLCQDCYIDINR) (7-82)  Cpn60_TCP1 (172-465)  PIP5K (1150-1373) | 1.20e-22  2.60e-10  4.70e-50 |
| GSPATT00004174001 | PtPIPK3b /III | scaffold_9 | 5/4 | [FYVE](http://smart.embl-heidelberg.de/smart/do_annotation.pl?DOMAIN=FYVE&TYPE=SMART&START=7&END=92&LENGTH=85&E_VALUE=1.0362410674924e-17&BLAST=LFTTKWVQDKEAKCCKKCSSQFKAIFRRKHHCRNCGGVFCDRYRFCHRTQNSCSNFFIDKTNFKNYQEIKKNKVRLCQDCFNDISK) (7-92)  Cpn60_TCP1 (170-463)  PIP5K (1163-1386) | 1.04e-17  3.80e-10  2.40e-49 |
| GSPATT00033281001 | PtPIPK3 /III c | scaffold_136 | 5/4 | [FYVE](http://smart.embl-heidelberg.de/smart/do_annotation.pl?DOMAIN=FYVE&TYPE=SMART&START=10&END=82&LENGTH=72&E_VALUE=4.37545050204667e-16&BLAST=IKRDKWKAESKVTKCEHCDRQFYYLFRTKHHCRKCGLVFCSDCSSNFIDGIHFAQNTEKKVRLCGVCYDQVLK) (10-82)  Cpn60_TCP1 (163-462)  PIP5K (1227-1453) | 4.38e-16  7.40e-16  2.70e-49 |
| GSPATT00022539001 | PtPIPK3d /III | scaffold_74 | 6/5 | [FYVE](http://smart.embl-heidelberg.de/smart/do_annotation.pl?DOMAIN=FYVE&TYPE=SMART&START=10&END=80&LENGTH=70&E_VALUE=6.14661054769728e-11&BLAST=LSRDKWKPESKVTKCEHCDRQFYYLFRAKHHCRKCGLVFCSDTNFIDGLHFAQNTEKKVRLCGVCYDQVLK) (10-80)  Cpn60_TCP1 (160-454)  PIP5K (1217-1443) | 6.15e-11  2.10e-16  3.50e-49 |
| GSPATT00015691001 | PtPIPK3e /III | scaffold_45 | 5/4 | [FYVE](http://smart.embl-heidelberg.de/smart/do_annotation.pl?DOMAIN=FYVE&TYPE=SMART&START=15&END=85&LENGTH=70&E_VALUE=2.28979090007644e-19&BLAST=IPKKKWKEDQEINQCGMCDNKFNALFRRKHHCRRCGYIFCQDCSNHFIDGPPNEGEKKIRFCRRCYDKINQ) (15-85)  Cpn60_TCP1 (149-446)  PIP5K (1140-1367) | 2.29e-19  1.50e-12  1.90e-47 |
| GSPATT00012283001 | PtPIPK3f /III | scaffold_33 | 4/3 | [FYVE](http://smart.embl-heidelberg.de/smart/do_annotation.pl?DOMAIN=FYVE&TYPE=SMART&START=15&END=85&LENGTH=70&E_VALUE=2.28979090007644e-19&BLAST=IPKKKWKEDQEINQCGMCDNKFNALFRRKHHCRRCGYIFCQDCSNHFIDGPPNEGEKKIRFCRRCYDKINQ) (28-56)*^2^  Cpn60_TCP1 (129-417)  PIP5K (1111-1338) | 2.00e-9  1.10e-13  3.10e-46 |
| GSPATT00013442001 | PtPIPK4a | scaffold_38 | 1/- | PIP5K (64-320) | 1.30e-38 |

*^1^ TMs, transmembrane regions

*^2^ BLAST

*^3^ Three PIPK genes are recovered in the PFAM database but are absent from the final data set. The gene locus tags are GSPATT00008048001, GSPATT00031424001 and GSPATT00034475001. GSPATT00008048001 was recovered in our initial screen, seems to code for a PtPIPK4a-like protein, yet, our phylogenetic analysis had placed it in the PIPK2 group. Most probably, however, it is a pseudogene (L. Sperling and O. Arnaiz, personal communication). GSPATT00031424001 and GSPATT00034475001 code for transmembrane PIPK2-like genes, but the e-value for the PIPKc domain is very low (10^-8^-10^-7^).
