# Supplementary material for: Genome-Wide Analysis of the Phosphoinositide Kinome from Two Ciliates Reveals Novel Evolutionary Links for Phosphoinositide Kinases in Eukaryotic Cells
Source: PLoS One. 2013 Nov 11;8(11):e78848. doi: 10.1371/journal.pone.0078848 (PMC3823935; doi:10.1371/journal.pone.0078848)
Supplement: Table S3 — Eukaryotic PIPKs used for alignments and phylogenetic trees. (DOC) [file pone.0078848.s007.doc]

| **Organism**  Table S3. Eukaryotic PIPKs used for alignments and phylogenetic trees | **Accesion number** | **Gene name/comments** |
| --- | --- | --- |
| *Monosiga brevicollis* * | XP_001746418.1 | MbPIPK1 |
|  | XP_001745820.1 | MbPIPK2 / type I |
|  | XP_001745617.1 | MbPIPK3 / type II |
|  | XP_001744065.1 | MbPIPK4 / type IV |
|  | XP_001746222.1 | MbPIPK5 / type III |
| *Schizosaccharomyces pombe* | NP_594429.1 | SpIts3 |
|  | NP_596090.2 | SpFab1 |
| *Candida albicans* | XP_710436.1 | CaMss4 |
|  | XP_722558.1| | CaFab1 |
| *Caenorhabditis elegans* | NP_497500.1 | CePPK-2 / type II |
|  | NP_491576.2 | CePPK-1 / type I |
|  | NP_510155.3 | CePPK-3 / type III |
| *Dictyostelium discoideum* | XP_647148.1 | DDB_G0267588 |
|  | XP_641291.2 | DDB_G0280377 |
|  | XP_643612.1 | PIPKinA/PIK6 |
|  | XP_637938.2 | DdPI4P5Ka |
|  | XP_645021.2 | DdPI4P5Kb / pseudoPIPK |
|  | AAP13090.1 | DdRpkA / type IV |
|  | XP_001732989.1 | DdPIP5K3 / type III |
| *Saccharomyces cerevisiae* | CAA92347.1| | ScMss4 |
|  | P34756.1 | ScFAB1 |
| *Mus musculus* | NP_032871.2| | MmPIPKIIα |
|  | AAH47282.1 | MmPIPKIIβ |
|  | NP_473438.1 | MmPIPKIIγ |
|  | BAA13030.1 | MmPIPKIα |
|  | EDL38772.1 | MmPIPKIβ |
|  | NP_032870.1 | MmPIPKΙγ |
|  | NP_035216.2 | MmPIKfyve |
| *Arabidopsis thaliana* | NP_192028.1 | AtPIPK10 |
|  | Q8L850.2 | AtPIPK9 |
|  | Q56YP2.1 | AtPIPK1 |
|  | NP_001078484.4 | AtPIPKIII |
| *Oryza sativa* | BAD08735.1 | OsFab1-like |
| *Chlamydomonas reinhardtii* | XP_001696909.1 | CrFab1-like |
|  | XP_001697577.1 | CrPIPK1 |
|  | XP_001696663.1 | CrPIPKL |
| *Trypanosoma cruzi* | XP_817303.1 | TcPI4P5Ka |
|  | XP_813452.1 | TcPI4P5Kb |
|  | XP_819626.1 | TcPI4P5Kc / pseudoPIPK |
|  | XP_809743.1 | TcPI4P5Kd / type II-like |
|  | XP_804543.1 | TcPIPKIII |
| *|Trypanosoma brucei* | XP_822637.1 | TbPIPK / pseudoPIPK |
| *Leishmania major* | XP_001686413.1 | LmPI4P5Ka |
|  | XP_843165.1 | LmPI4P5Kb |
|  | XP_001686607.1 | LmPI4P5Kc / type II-like |
|  | XP_848043.1 | LmPIPKIII |
| *Leishmania infantum* | XP_001468872.1 | LiPI4P5K |
| *|Leishmania braziliensis* | XP_001568671.1 | LbPIPK/ pseudoPIPK |
| *Phytophthora infestans* | EEY64779.1 | PhyPIPK-C |
|  | EEY64612.1 | PhyPIPK-D11 / type IV / pseudoPIPK |
|  | EEY57162.1 | PhyPIPK-D2 / type IV |
|  | EEY57873.1 | PhyPIPK-D5 / type IV |
|  | EEY54682.1 | PhyPIPK-D12 / type IV / pseudoPIPK |
|  | EEY58255.1 | PhyPIPK-D9 / type IV |
|  | EEY64054.1 | PhyPIPKD10 / type IV |
|  | EEY69098.1 | PhyPIPK-D1 / type IV |
| *Giardia intestinalis* | EES98657.1 | GiPIPK |
| *Giardia lamblia* | EDO82343.1 | GlPIPK |
|  | EFO64172.1 | GlPIPKL |
| *Trichomonas vaginalis* | XP_001579530.1 | TvPIPK1 |
|  | XP_001583013.1 | TvPIPKL / putative type III |
| *Cryptosporidium parvum* | XP_001388435.1 | CpPIPK |
| *Cryptosporidium muris* | EEA05378.1 | CmPIPK1 |
| *Babesia bovis* | XP_001610265.1 | BbPIPK1 |
|  | XP_001612211.1 | BbPIPKL / putative type III |
| *Toxoplasma gondii* | EEE27003.1 | TgPIPK1 |
|  | EEE25461.1 | TgPIPKL / putative type III |
| *Perkinsus marinus* | EER05356.1 | PmPIPK1 |
| *Theileria parva* | XP_764765.1 | TpPIPK1 |
|  | XP_764623.1 | TpPIPKL / putative type III |
| *Plasmodium falciparum* | XP_001351035.1 | PfPIPK1 |
|  | XP_001348296.1 | PfPIPKL / putative type III |
| *Naegleria gruberi* * | XP_002670481.1 | NgPIPK5 |
|  | XP_002680330.1 | NgPIPK4 / type II-like |
|  | XP_002670454.1 | NgPIPK1 |
|  | XP_002683386.1 | NgPIPK2 |
|  | XP_002682289.1 | NgPIPK3 / putative type III |

* *Monosiga brevicolis* and *Naegleria gruberi* PIPKs were numbered arbitrarily

Comments concerning the PIPK cohort and the tree in Fig. 5: (i) most *Dictyostelium discoideum* PIPK genes (DdPIPKa, DdPIK6, DDBG0280, DDBG0267 and DdRpkA) were largely unrelated to the identified groups and occupied different positions in the tree with the striking exception of DdPIP5K3, the PIPKIII/FAB1 ortholog; (iii) 3 *Monosiga brevicolis* PIPKs were consistently assigned to type I (MbPIPK2), type II (MbPIPK3) and type III (MbPIPK5) types while 2 additional PIPKs (MbPIPK1, a PH- and FYVE-domain containing PIPK, and MbPIPK4, a type IV PIPK) were unrelated to the identified groups and occupied different positions in the tree. These are not included in the tree in Fig 5; (iii) The tree does not include an additional number of PIPKs (12 sequences) that were originally included in the data set but were subsequently eliminated since they exhibited inconsistent phylogenetic relationships in multiple trial neighbor-joining trees utilizing different amino acid substitution models (for some PIPKs this was evident by long branch lengths). This eliminated set of PIPKs included mostly pseudo-PIPKs. (iv) *Naegleria* PIPKs were most related to PIPKs from excavates, but NgPIPK3, a FYVE and Cpn60_TCP1 domain-containing PIPK was unambiguously assigned to the PIPKIII branch (not shown in Fig. 5).
